# Coral taxonomy and local stressors drive bleaching prevalence across the Hawaiian Archipelago in 2019

**DOI:** 10.1371/journal.pone.0269068

**Published:** 2022-09-01

**Authors:** Morgan Winston, Thomas Oliver, Courtney Couch, Mary K. Donovan, Gregory P. Asner, Eric Conklin, Kimberly Fuller, Bryant W. Grady, Brittany Huntington, Kazuki Kageyama, Tye L. Kindinger, Kelly Kozar, Lindsey Kramer, Tatiana Martinez, Amanda McCutcheon, Sheila McKenna, Ku‘ulei Rodgers, Cameron Kaʻilikea Shayler, Bernardo Vargas-Angel, Brian Zgliczynski

**Affiliations:** 1 Cooperative Institute for Marine and Atmospheric Research, University of Hawai‘i, Honolulu, Hawai‘i, United States of America; 2 Pacific Islands Fisheries Science Center, National Marine Fisheries Service, Honolulu, Hawai‘i, United States of America; 3 Center for Global Discovery and Conservation Science and School of Geographic Sciences and Urban Planning, Arizona State University, Hilo, Hawai‘i, United States of America; 4 The Nature Conservancy, Honolulu, Hawai‘i, United States of America; 5 Division of Aquatic Resources (O‘ahu), Anuenue Fisheries Research Center, Honolulu, Hawai‘i, United States of America; 6 Pacific Island Network Inventory and Monitoring Program, Hawai‘i National Park, Hawai‘i, United States of America; 7 Division of Aquatic Resources (Kona), Kailua-Kona, Hawai‘i, United States of America; 8 Division of Aquatic Resources (Maui), Wailuku, Hawai‘i, United States of America; 9 Hawai‘i Institute of Marine Biology, Kāne‘ohe, Hawai‘i, United States of America; 10 Division of Aquatic Resources, Kaua‘i, Līhuʻe, Hawai‘i, United States of America; 11 Scripps Institution of Oceanography, La Jolla, California, United States of America; Biodiversity Research Center, TAIWAN

## Abstract

The Hawaiian Archipelago experienced a moderate bleaching event in 2019—the third major bleaching event over a 6-year period to impact the islands. In response, the Hawai‘i Coral Bleaching Collaborative (HCBC) conducted 2,177 coral bleaching surveys across the Hawaiian Archipelago. The HCBC was established to coordinate bleaching monitoring efforts across the state between academic institutions, non-governmental organizations, and governmental agencies to facilitate data sharing and provide management recommendations. In 2019, the goals of this unique partnership were to: 1) assess the spatial and temporal patterns of thermal stress; 2) examine taxa-level patterns in bleaching susceptibility; 3) quantify spatial variation in bleaching extent; 4) compare 2019 patterns to those of prior bleaching events; 5) identify predictors of bleaching in 2019; and 6) explore site-specific management strategies to mitigate future bleaching events. Both acute thermal stress and bleaching in 2019 were less severe overall compared to the last major marine heatwave events in 2014 and 2015. Bleaching observed was highly site- and taxon-specific, driven by the susceptibility of remaining coral assemblages whose structure was likely shaped by previous bleaching and subsequent mortality. A suite of environmental and anthropogenic predictors was significantly correlated with observed bleaching in 2019. Acute environmental stressors, such as temperature and surface light, were equally important as previous conditions (e.g. historical thermal stress and historical bleaching) in accounting for variation in bleaching during the 2019 event. We found little evidence for acclimation by reefs to thermal stress in the main Hawaiian Islands. Moreover, our findings illustrate how detrimental effects of local anthropogenic stressors, such as tourism and urban run-off, may be exacerbated under high thermal stress. In light of the forecasted increase in severity and frequency of bleaching events, future mitigation of both local and global stressors is a high priority for the future of corals in Hawai‘i.

## Introduction

Coral bleaching driven by climate-induced marine heatwaves stands as one of the single greatest threats to coral reefs [1]. As the pace of warming ocean temperatures have risen, so too have the frequency and severity of mass coral bleaching events around the world [2, 3]. Over a prolonged period ranging from 4 to 6 weeks, exposure to elevated temperatures 1°C above the local average temperature of the warmest month of the year can often trigger bleaching due to thermal stress [4], thereby breaking down the symbiotic relationship between corals and the dinoflagellate algae living within their tissues. After disruption of this symbiosis, corals typically have been exposed to significant oxidative stress [5–7], lack a crucial energy source [8], and become increasingly vulnerable to disease [9, 10]. Severe bleaching can result in widespread and immediate partial or full mortality of coral colonies [11, 12]. For those corals that survive, the sub-lethal effects of bleaching may interrupt processes of growth and reproduction [4, 13, 14]. As coral reefs worldwide face a barrage of threats imposed by a rapidly warming climate, further understanding of what drives bleaching and how local environmental factors interplay with heat stress to differentially affect coral communities is critical for predicting the state of future reefs. Identifying bleaching resistant coral taxa and reef assemblages can inform local management targets with the goal of supporting coral resilience following repeated bleaching events.

The extent of bleaching across reefs in response to thermal stress is often variable and can be a function of local conditions [15]. Site- and region-specific factors including water flow, weather patterns, irradiance, and community structure [16–20], as well as anthropogenic disturbances due to sedimentation and nutrient enrichment associated with land-based sources [21, 22], can impact bleaching extent and severity. Coral taxa exhibit marked variability in bleaching susceptibility. Certain species are able to withstand repeated thermal stress while others are unable to recover after a single bleaching episode [23–26]. Fast growing, branching taxa typically bleach rapidly and undergo whole colony mortality, while slower growing, massive taxa may take longer to bleach and display increased survivorship rates despite remaining bleached for longer time periods [24]. With continued warming, these hardier and more slowly growing taxa are hypothesized to replace weedy, fast-growing taxa on future reefs [24, 27]. While bleaching susceptibility is largely dependent on colony morphology, growth rate, reproduction, and overall life history strategies [28], bleaching response also varies between coral taxa due to differences in prior thermal stress exposure and acclimatization to particular thermal regimes [29–31]. Moreover, variation in bleaching susceptibility can also be influenced by the composition and characteristics of symbiotic algae residing within the host [32–34]. Growing evidence suggests that corals and their algal symbionts are capable of acclimatization and selective adaptation to thermal stress, resulting in greater bleaching resistance within populations [23, 29, 30, 35, 36].

Coral bleaching events in the Hawaiian Archipelago have increased in frequency and severity since 1996 [19, 37–39]. In 2014, the Northwestern Hawaiian Islands (NWHI) experienced severe thermal stress and high levels of bleaching resulting in loss of coral cover reaching 68% at some sites [39]. During the 2015 thermal stress event in the main Hawaiian Islands (MHI), catastrophic bleaching was observed, with up to a 71% loss in coral cover on the west coast of Hawaiʻi Island [38], relative to pre-bleaching values, and close to 50% in both Kāne‘ohe and Hanauma Bay on the island of O‘ahu [17, 40, 41]. In the fall of 2019, the MHI experienced another marine heatwave with some portions of the MHI and NWHI experiencing more than 20 weeks of accumulated thermal stress, resulting in the third major bleaching event recorded in the Hawaiian Islands within a 6-year period.

The Hawai‘i Coral Bleaching Collaborative (HCBC) was established following the 2014 bleaching event and includes academic, non-governmental and governmental partners active in research, restoration, conservation, and management of coral reef resources in Hawai‘i. The primary mission of the HCBC is to coordinate coral bleaching surveys across the state to monitor the extent and severity of mass bleaching events, collate and share data about these events to understand their impact, and develop management recommendations for reducing impacts of future events. To document the 2019 bleaching event, HCBC launched a large multi-institutional response consisting of diver visual assessments and image-based surveys across the Hawaiian Archipelago. The goals of this study were to: 1) assess the spatial and temporal patterns of thermal stress; 2) examine taxa-level patterns in bleaching susceptibility; 3) quantify spatial variation in bleaching extent; 4) compare 2019 patterns to those recorded during the 2014 and 2015 bleaching events; 5) determine key natural and anthropogenic predictors (hereafter referred to as drivers) of bleaching in 2019; and 6) explore site-specific management strategies to mitigate future bleaching events using a scenario-based sensitivity analysis.

## Methods and materials

### Coral bleaching surveys

A total of 46 surveys were conducted across four of the NWHI (Kure Atoll [Hōlanikū], Pearl and Hermes Atoll [Manawai], Lisianski Island [Kapou], French Frigate Shoals [Lalo]) from 27 August to 4 September 2019 (S1 Fig). A total of 2,131 surveys were conducted at six of the MHI (Kaua‘i, Oʻahu, Moloka‘i, Maui, Lānaʻi, and Hawaiʻi Island) from 20 August to 7 December 2019 (S1 Table, S2 Fig). Surveys in the NWHI were opportunistically conducted during a scheduled National Oceanic and Atmospheric Administration (NOAA) National Coral Reef Monitoring Program (NCRMP) field mission, which preceded the peak of thermal stress from late September through October.

Surveys were conducted across three depth bins (shallow [0–6 m], mid [>6–18 m], and deep [>18–30 m]) per island. Survey locations were also selected based on a range of predicted thermal stress, status as long-term monitoring sites, and accessibility. Across the institutions that participated in coral bleaching surveys, three different yet complementary survey approaches [42] were employed that provided similar ecological outputs: *in situ* diver rapid visual surveys, photoquadrat surveys, and transect-intercept surveys (see S2 Table and [43] for detailed methods and data access). Photoquadrat images were analyzed using the computer-based software PhotoGrid 1.0 or web-based software CoralNet [44]. The following data were recorded or calculated by each survey approach: total live coral cover (%) and percent of the total coral cover that was bleached (hereafter referred to as percent bleached), and the absolute percent cover and percent of the taxa cover that was bleached for up to eight of the most dominant coral taxa per site.

The relative taxonomic bleaching susceptibility was also calculated per survey when possible. All taxa were assigned a score from (1) least susceptible to (5) most susceptible to bleaching using a combination of taxon-specific percent bleached values and bleaching severity in 2019, scores developed in previous analyses [39], and unpublished data from NOAA Pacific Islands Fisheries Science Center’s Ecosystem Sciences Division. The relative taxonomic bleaching susceptibility (BS) was calculated for each surveyed site by the following equation:

$$BS=(\sum_{t=1}^{T_{s}}{P_{st\;}S}_{t})/P_{s}$$

where *t* is taxon, *T*_*s*_ is the total number of taxa for a given survey *s*, *P*_*st*_ is the percent cover of a given taxon for a given survey, *S*_*t*_ is the susceptibility score of a given taxon (S3 Table) and *P*_*s*_ is the percent cover of all live coral at a given survey.

To assess temporal trends, historical data on bleaching prevalence collected during 2014 (NWHI surveys) and 2015 (MHI surveys) were used (see [39, 45] for details).

### Statistical analysis

All data were analyzed using R Statistical Software 3.6.3 [46]. Code base is well developed and available upon request to the corresponding author. To assess variation in thermal stress over time, NOAA Coral Reef Watch (CRW) daily 5-km sea surface temperature (SST) and Degree Heating Week (DHW) (a metric of thermal stress accumulation used to predict bleaching) version 3.1 satellite data were used [47, 48]. CRW calculates DHW (°C-weeks) as the accumulation of instantaneous bleaching heat stress, or HotSpots, during the most recent 12-week period [49]. HotSpots are defined as positive SST anomalies above the Maximum of the Monthly Mean SST climatology (MMM), or upper tolerance threshold for corals. Overall, when the DHW value reaches 4°C-weeks, significant coral bleaching typically occurs; at 8 DHW and higher, severe and widespread bleaching and mortality is predicted. Data were extracted from a 6-km buffer surrounding the shoreline of each island/atoll that was surveyed in 2019, and daily mean estimates were derived and used to generate time-series per island/atoll to visually assess long-term changes in temperature and DHW from 1985 to 2019.

Taxa-level percent bleaching data in 2019 were only collected at four of the MHI (O‘ahu, Lānaʻi, Maui, and Hawaiʻi Island) and unevenly across depth strata (S4 Table). Non-metric multidimensional scaling (nMDS) was conducted using square-root transformed Bray-Curtis dissimilarity matrices of relative live coral cover to visualize community level differences in percent bleached and taxonomic susceptibility between islands (‘metaMDS’ function, *vegan* package [50]. A single site dominated by *Porites monticulosa* was removed because it strongly skewed community composition. To determine how the bleaching response differed between dominant coral taxa, data for species observed on less than six surveys per island were removed prior to generating visual representations of mean percent bleaching per taxa. Following Fenner 2005 [51], *Porites lutea* was considered a synonym of *Porites evermanni*; therefore, all observations of *P*. *evermanni* were considered to be *P*. *lutea*.

To assess spatial and temporal patterns as well as investigate potential drivers of bleaching, we first addressed spatial auto-correlation (i.e. the possibility that near-by sites share similar patterns in bleaching) among surveys by performing hierarchical clustering. Surveys within 1 km of one another, irrespective of depth, were assigned distinct cluster identifications [52]; the number of surveys in these resulting clusters ranged from 1 to 81. Within each of these clusters, surveys conducted in each depth bin (shallow, mid, or deep) were further combined to calculate a single mean value of percent bleached per depth bin (S5–S7 Tables). Given the large island size, clusters in the MHI were assigned to sub-island zones (e.g. North, South, West, etc.) to test for intra-island variability in bleaching (see [45] for details and data access). Zone boundaries were chosen using a combination of personal observations and sectors identified to be spatially homogeneous based on long-term benthic cover data [53]. If there were less than three mean estimates of % bleached in a depth bin across all replicate clusters within a given zone, then that depth bin was dropped from that zone altogether in subsequent analyses.

To account for the variability in surveys within a cluster, a weighting factor was assigned to each cluster according to the amount of information and variation each cluster provided to the overall inference. Weighting factors were calculated as the inverse of the standard error of mean percent bleaching. They were then transformed and scaled prior to analysis by the following process: weighting factors greater than the 95th percentile were set to the 95th percentile value, all weighting factors were then divided by the 95th percentile to result in an overall range of weights from > 0 to 1, and weighting factors that were NA (due to n = 1 within the depth bin/cluster) were set to the 5th percentile. All spatial, temporal, and driver analyses used weighting factors to account for the variability in sample sizes per cluster.

The response variable (percent bleached) was square-root transformed to meet visual assumptions of normality for the spatial, temporal, and drivers analyses. Spatial differences in % bleached in 2019 were compared across the Hawaiian Archipelago at the finest scale possible (zone in the MHI; island/atoll in the NWHI) using a weighted one-way ANOVA followed by Tukey’s post hoc tests. Data were only included in the temporal analysis for depth bins and zones (MHI) or islands (NWHI) that were visited in both 2014 (NWHI only) or 2015 (MHI only) and 2019. The 2014 and 2015 data sets were clustered and weighted following the same approach described above for consistency with the 2019 data set and to account for spatial autocorrelation. Temporal differences in % bleached between 2015 and 2019 in the MHI were assessed using a weighted linear mixed model (LMM) approach. Likelihood ratio tests (LRT), following tests for normality and equal variances, were used to determine the significance of year, zone, and their interaction, with island as a random effect. If zone was significant, Tukey post hoc analyses were used to determine which zones in the MHI were significantly different between years. Differences in % bleached between 2014 and 2019 in the NWHI were examined using a weighted two-way ANOVA with fixed effects of year, island, and their interaction.

Potential natural and anthropogenic drivers of the 2019 bleaching event were investigated using a weighted linear model approach that assessed a number of hypothesized mechanisms associated with bleaching (S8 Table). Interaction terms were also included to examine how each potential driver modified the relationship between acute thermal stress and bleaching, and taxonomic susceptibility and bleaching. Observations of bleaching from deep (> 18 m) sites were excluded from this analysis based on poor sampling distribution across this depth range. Surface light (PAR) and light attenuation (kdPAR) raster data derived from satellite imagery was masked at the pixel-level as a quality control measure. We overlaid the ocean color rasters with bathymetry data. Pixels that had an area greater than 5% registered in depths of 30 m or shallower were masked. We then derived the ocean color values (PAR and kdPAR) from an extraction of the nearest pixel. Clusters were removed from the analysis if NA values for any predictor variables were detected. Each predictor was transformed as needed to improve normality (S9 Table) and then standardized and centered prior to analysis. Backwards step-wise model selection was performed using Bayesian information criterion (BIC) with the stepAIC function from the R package ‘MASS’ [54] to identify the best-fit model, with square-root transformed percent bleaching in 2019 as the response variable. Partial regression plots were generated to visualize the effects of each driver variable on predicted bleaching while accounting for the effects of all other drivers. Interaction terms were visualized using prediction interaction surface plots, where predicted bleaching was calculated holding all variables constant at their means except for the two interacting variables of interest, which were varied over their observed ranges. To further understand the impact of each driver variable on predicted bleaching, we perturbed the model by increasing and decreasing each driver in turn by one standard deviation (SD) while holding all other drivers constant at their mean. Mean predicted bleaching per variable perturbation was calculated and compared.

To investigate potential management actions that may mitigate future bleaching events in the main Hawaiian Islands, we used scenario-based modelling to simulate a heating event by holding acute thermal stress constant at the 95th percentile observed during the 2019 event (10.5 DHW). Using the best-fit model, we perturbed a subset of drivers (that could potentially be manipulated by managers and including surface light [PAR], sewage effluent, urban run-off, taxonomic susceptibility, and tourism) in turn by reducing each variable by one SD and then generating bleaching predictions at the depth bin per cluster level. To identify what management action would be most effective at reducing bleaching, we calculated the difference between the predicted bleaching under these conditions to the original model predictions.

## Results

### Temporal and geographic trends in long-term thermal stress

**Fig 1:**
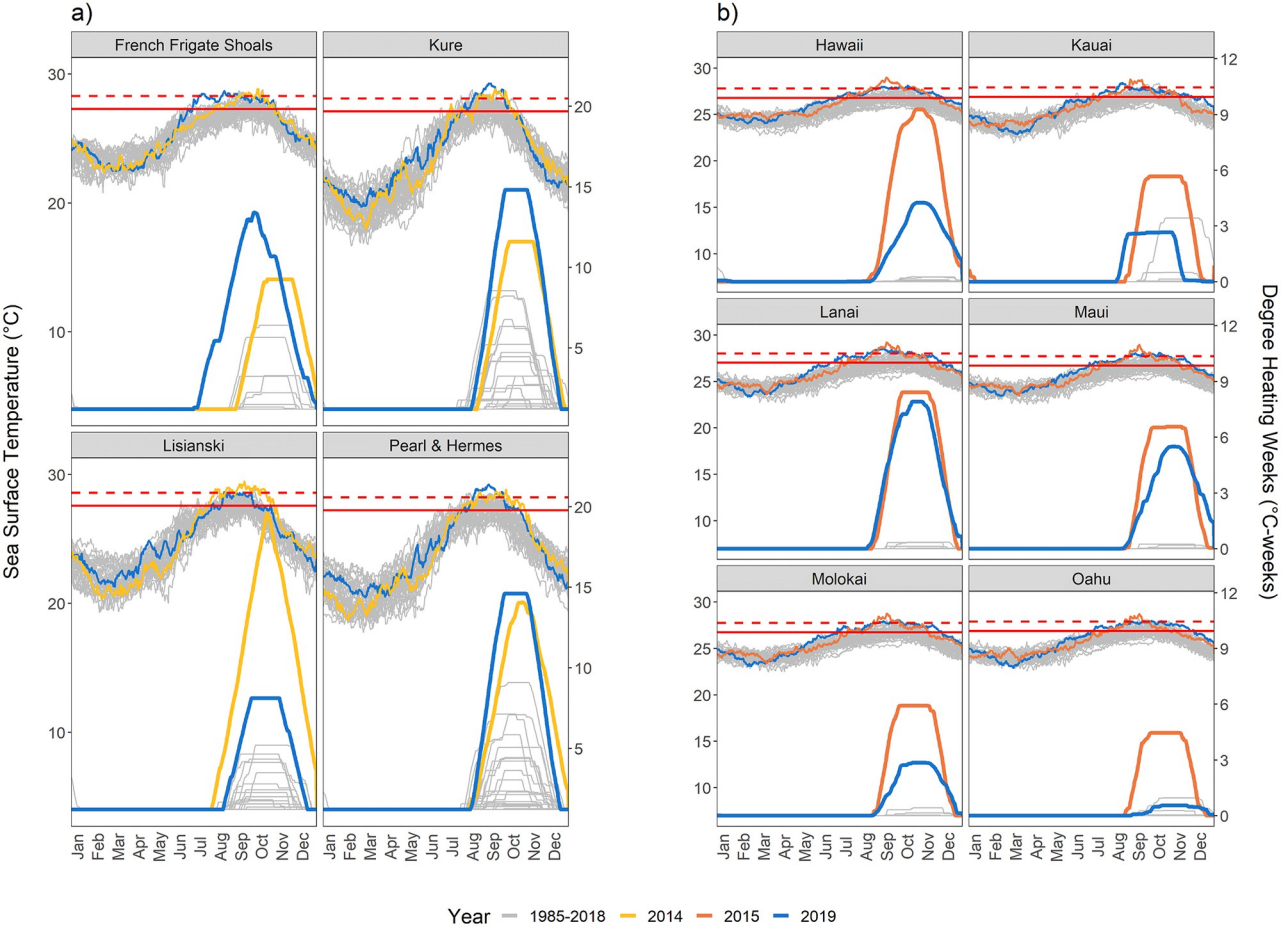
Sea surface temperature (SST) (top of panel) and NOAA Coral Reef Watch’s Degree Heating Weeks (DHW) data (bottom of panel) for the (a) Northwestern Hawaiian Islands (French Frigate Shoals, Kure Atoll, Lisianski Island, and Pearl and Hermes Atoll) and (b) Main Hawaiian Islands (Kaua‘i, O‘ahu, Moloka‘i, Maui, Lānaʻi, and Hawai‘i Island). Solid horizontal red line: the maximum of monthly mean SST climatology (MMM); dashed horizontal red line: bleaching threshold SST (1ºC above MMM). Yellow, orange and blue lines represent temperature and DHW trends from 2014, 2015, and 2019, respectively; gray lines represent all other years from 1985–2018.

In the Northwestern Hawaiian Islands, Kure, Pearl and Hermes Atoll (PHR), and French Frigate Shoals (FFS) all experienced higher DHW in 2019 than in 2014, with DHW surpassing 12°C-weeks (Fig 1a). In contrast, thermal stress at Lisianski was far lower in 2019 (6.9°C-weeks) than in 2014 (18.4°C-weeks) and remained below the bleaching threshold unlike in 2014. The SSTs at Kure and PHR tracked above the bleaching threshold, reaching higher temperatures sooner and for a longer duration in 2019 than in 2014. SST at FFS also reached the MMM sooner in 2019 than in 2014, yet plateaued near the bleaching threshold.

Each of the main Hawaiian Islands experienced a longer duration and higher intensity of thermal stress during the 2015 bleaching event than during the 2019 bleaching event (Fig 1b). The maximum DHW experienced per island was 1.1–8.2 times higher in 2015 compared to the 2019. In late spring of 2019, SSTs across the MHI began to track well above historical records. The SST in 2019 plateaued at or close to the bleaching threshold, defined as 1ºC above the MMM climatology (a measurement of the upper limit for typical temperatures), while in 2015 it surpassed the bleaching threshold in the MHI. Peak thermal stress occurred within October in both years. O‘ahu experienced the lowest DHW of all the MHIs in both bleaching event years, with a maximum of 4.5 and 0.5°C-weeks in 2015 and 2019, respectively. Hawai‘i Island had the greatest difference in DHW between years, dropping from 9.3°C-weeks in 2015 (the highest across all MHI that year) to 4.3°C-weeks in 2019.

### Variability in coral community assemblages and bleaching response across islands

**Fig 2:**
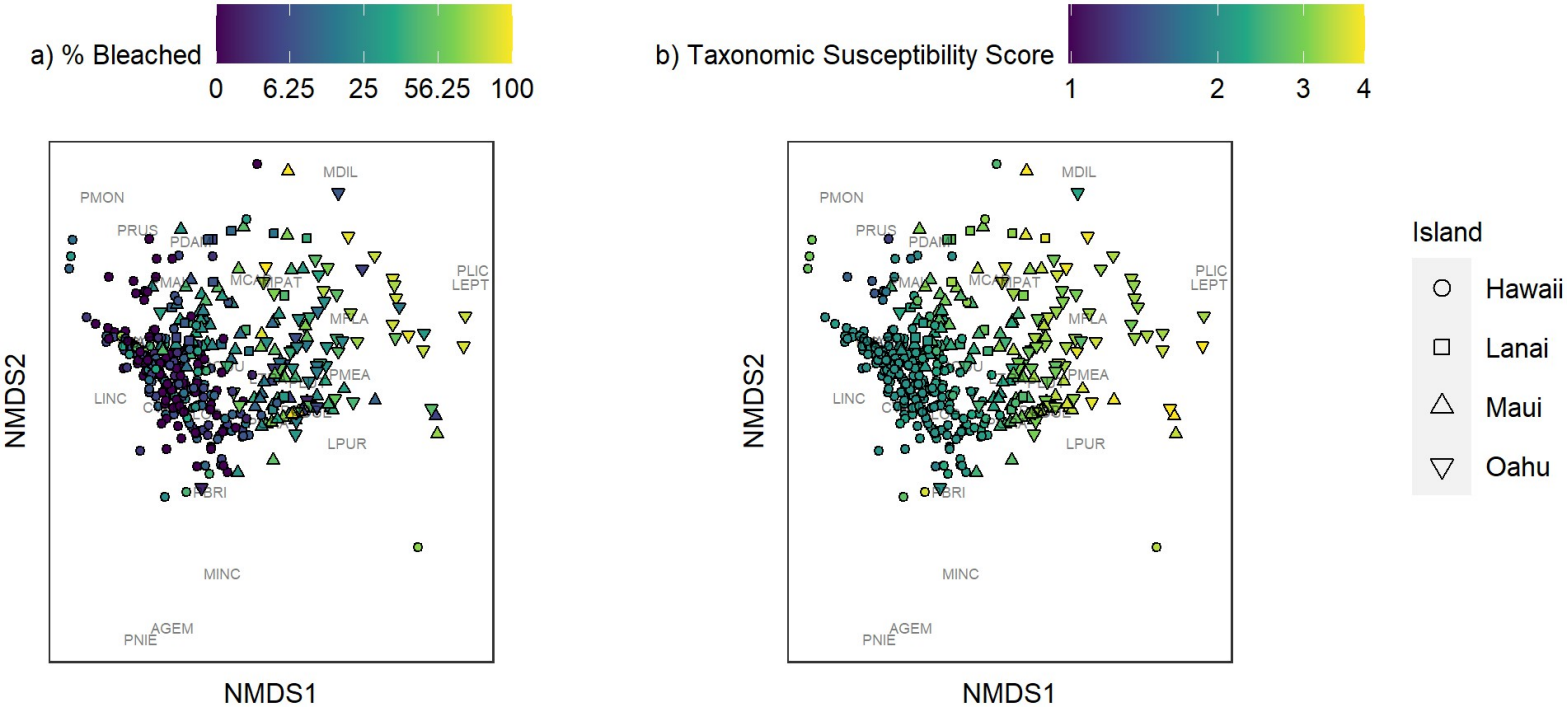
Two-dimensional ordination of the nonmetric multidimensional scaling (NMDS) configuration showing site scores based on Bray-Curtis dissimilarities of taxa-level relative live coral cover with overlaid percent bleached (a) and taxonomic susceptibility (b) recorded in 2019. Taxa scores are shown in black text (see S3 Table for code definitions). Points are shaped by island (MHI only). Stress score = 0.15. Species codes: AGEM = *Acropora gemmifera*, CVAU = *Cycloseris vaughani*, LPUR = *Leptastrea purpurea*, LEPT = *Leptastrea spp*., LINC = *Leptoseris incrustans*, MCAP = *Montipora capitata*, MDIL = *Montipora dilatata*, MFLA = *Montipora flabellata*, MINC = *Montipora incrassata*, MPAT = *Montipora patula*, PDUE = *Pavona duerdeni*, PMAL = *Pavona maldivensis*, PVAR = *Pavona varians*, PDAM = *Pocillopora damicornis*, PGRA = *Pocillopora grandis*, PMEA = *Pocillopora meandrina*, PBRI = *Porites brighami*, PCOM = *Porites compressa*, PODU = *Porites duerdeni*, PLIC = *Porites lichen*, PLOB = *Porites lobata*, PLUT = *Porites lutea*, PMON = *Porites monticulosa*, PRUS = *Porites rus*, POSP = *Porites spp*., PNIE = *Psammocora nierstraszi*.

**Fig 3:**
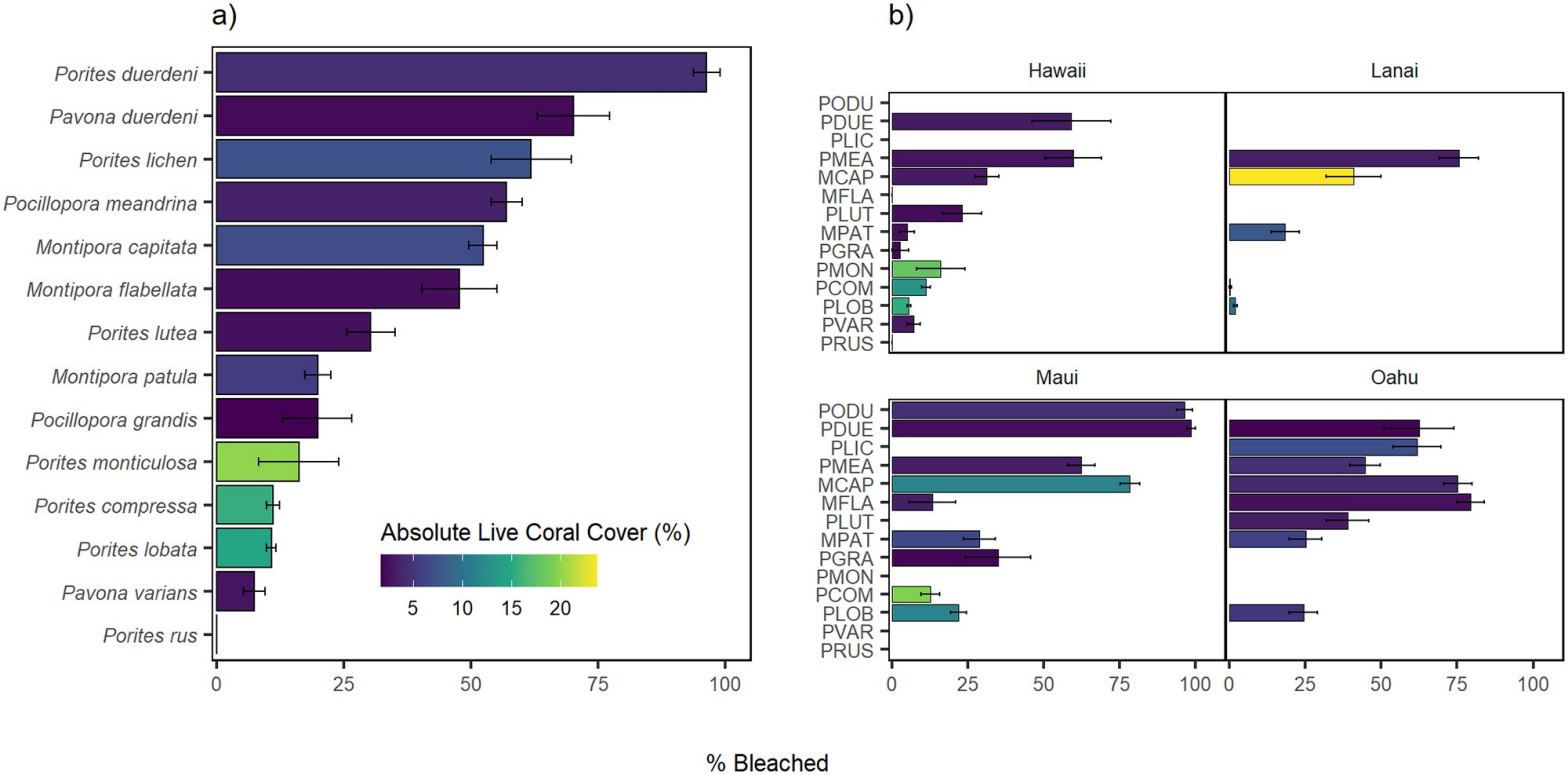
Species-level mean (± SE) percent bleached colored by absolute cover for all depths combined at the (a) domain level (MHI) and (b) island level in 2019. Gaps between bars represent taxa observed on fewer than six surveys per island.

The coral community structure in 2019 in the MHI appeared to follow a longitudinal shift, with Maui and Lānaʻi having more similar communities, while communities diverged the most between Hawai‘i Island and O‘ahu (Fig 2). The percent bleached and the relative susceptibility of corals to bleaching also appeared to vary most between Hawai‘i Island and O‘ahu, with greater levels of bleaching and more susceptible coral taxa observed on O‘ahu despite low total coral cover there (absolute individual species-level cover all fell below 7.5%, Fig 3).

The taxa with the highest percent of bleaching across all islands combined was *Porites duerdeni* (mean = 96.3 **±** 2.63%), which was only observed on Maui (Fig 3). No bleaching was observed in *Porites rus*, and *Pavona varians* had the lowest observable bleaching extent amongst all species (mean = 7.35 **±** 2.13%). Of all genera observed, *Pocillopora* spp. were the most vulnerable to bleaching, with an island-scale mean extent of 53.10 **±** 2.92%), while overall *Porites* spp. were the least susceptible across all genera observed (mean = 13.74 **±** 0.85%) and tended to have higher absolute cover than other genera island-wide. Trends in species-level bleaching held across space for certain taxa (e.g. *Pocillopora meandrina* exhibited >50% bleached among all islands), but varied across islands for others (e.g. *Porites lutea* and *Montipora flabellata*; Fig 3). On Lānaʻi, a high extent of bleaching was observed in *Pocillopora meandrina*, a species with low absolute coral cover, while only moderate bleaching was observed in *M*. *capitata*, a species with comparatively high absolute cover. More species were observed at Hawai‘i Island than any other island and these taxa exhibited a range of bleaching responses, although the species with the highest extents of bleaching had the lowest absolute cover (*P*. *meandrina* and *Pavona duerdeni*). Very high bleaching (> 75%) was observed in three species (*M*. *capitata*, *Porites duerdeni*, *Pavona duerdeni*) on Maui, which had similar magnitudes of response on other islands, although not as severe.

### Spatial patterns in bleaching in 2019

**Fig 4:**
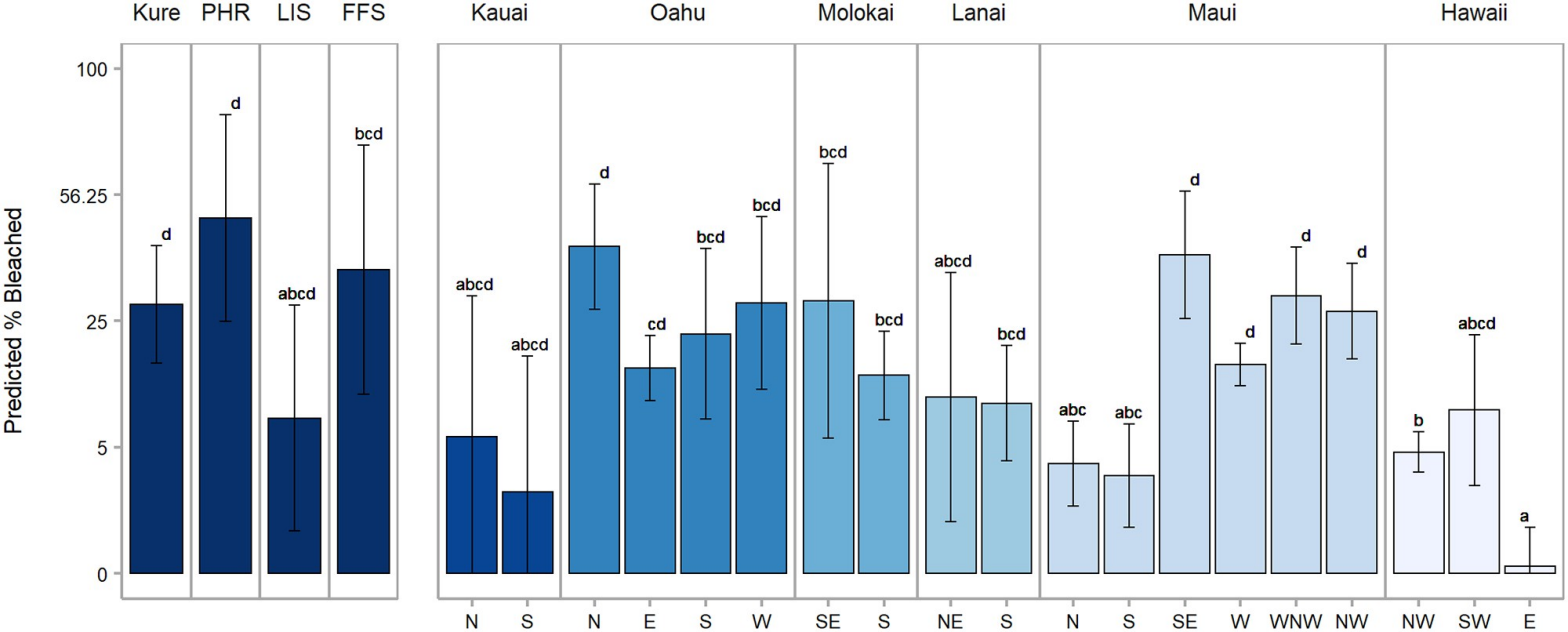
Predicted (± 95% CI) percent bleached per island (NWHI) and zone (MHI) for all depths combined in 2019. Zones are abbreviated in grey along the x-axis. Bars not connected by the same letter are significantly different at p < 0.05 based on Tukey post-hoc tests.

Bleaching varied significantly between locations across the archipelago (zone in the MHI and island/atoll in the NWHI; one-way ANOVA; F(_22,338_) = 10.92; p < 0.001). With low observed bleaching in east Hawai‘i Island (0.9%), specific significant differences occurred between this zone and multiple locations throughout both the MHI and NWHI (S3–S5 Figs). At the island level, Kaua‘i had the lowest percent bleaching observed across the entire archipelago (5.51 ± 1.66%), although bleaching at the surveyed zones here was not significantly different from other locations. While Kure and PHR had comparable levels of observed bleaching (34.7% and 34.4%, respectively), predicted bleaching was highest across the archipelago for PHR (S3 Fig). Within the NWHI, there was no significant difference in bleaching extent between islands/atolls, and low sample size precluded meaningful comparisons of bleaching across space at each island/atoll (S4 Fig). On O‘ahu, higher bleaching levels were recorded on northern reefs than elsewhere on the island, particularly on the northwest coastline (Fig 4). Conversely, the north and south zones of Maui experienced significantly less bleaching than other zones both within Maui and among the archipelago.

### Shifts in bleaching response over time

**Fig 5:**
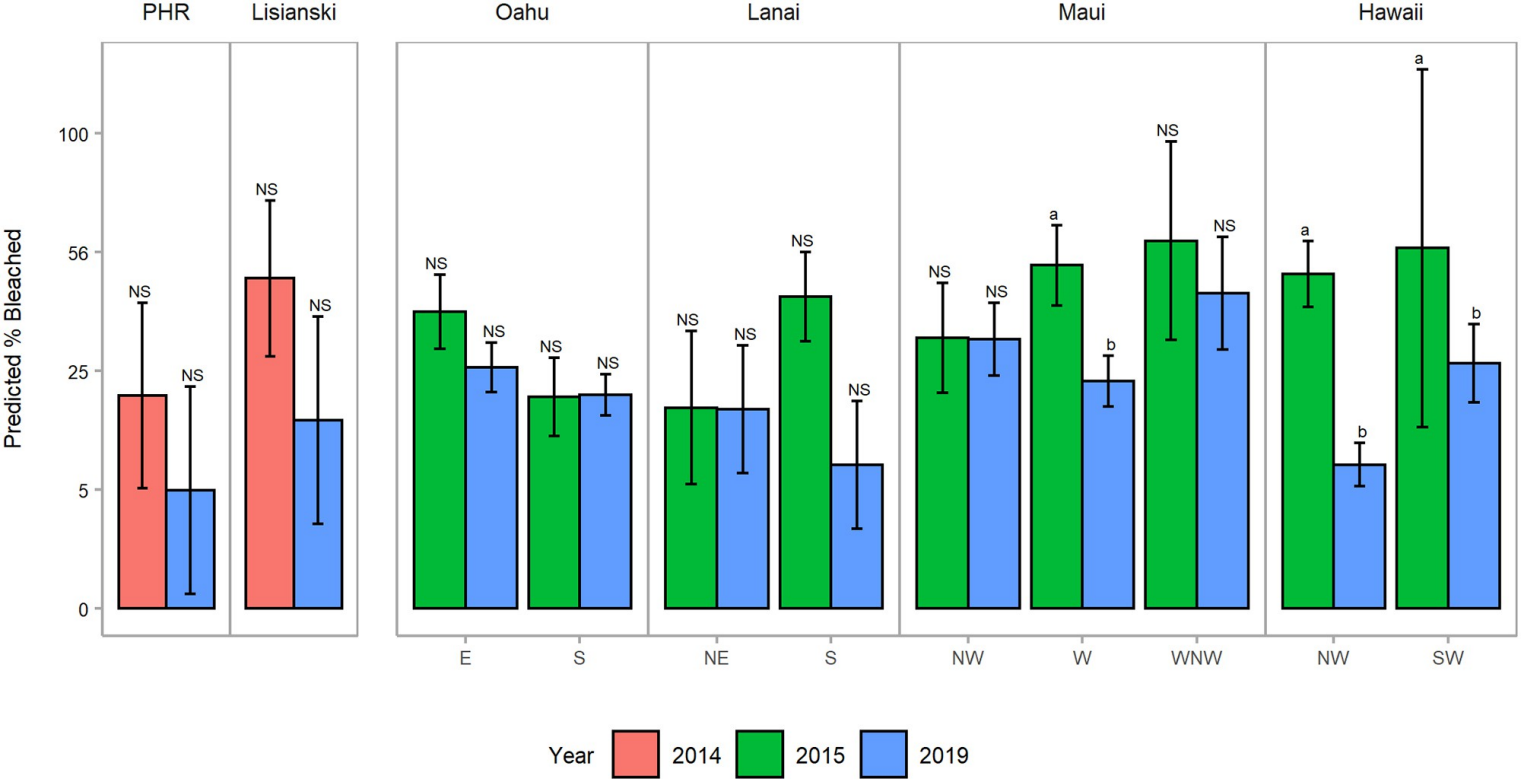
Predicted (± 95% CI) percent bleached per island and zone for all depths combined by year. Zones are abbreviated in grey along the x-axis. Islands or zones not connected by the same letter across years are significantly different at p < 0.05 based on Tukey post-hoc tests; ‘NS’ denotes non-significant differences.

Percent bleached varied significantly between the years 2015 and 2019 in the MHI overall (LRT: *χ*^2^ = 188.77, df = 1, p < 0.001), with a significant year x zone interaction (LRT: *χ*^2^ = 304.97, df = 17, p < 0.001) (S10 Table). At each of the MHI zones that were surveyed, the % bleached was either higher in 2015 or not significantly different than in 2019 (Fig 5, S6 and S7 Figs). Both zones along the leeward coast of Hawai‘i Island showed a significant difference in percent bleached between years, as did west Maui. O‘ahu had the lowest level of bleaching across islands in 2015 (29.74% ± 3.37 SE), and bleaching in 2019 was similar (25.76% ± 2.42 SE).

In the NWHI, % bleached varied significantly between islands (Two-way ANOVA; F(_1,16_) = 4.953; p = 0.041) and between years (F(_1,16_) = 8.262; p = 0.011). Pairwise post-hoc Tukey tests revealed no significant difference in bleaching between years at the island level.

### Drivers of the 2019 bleaching event

**Fig 6:**
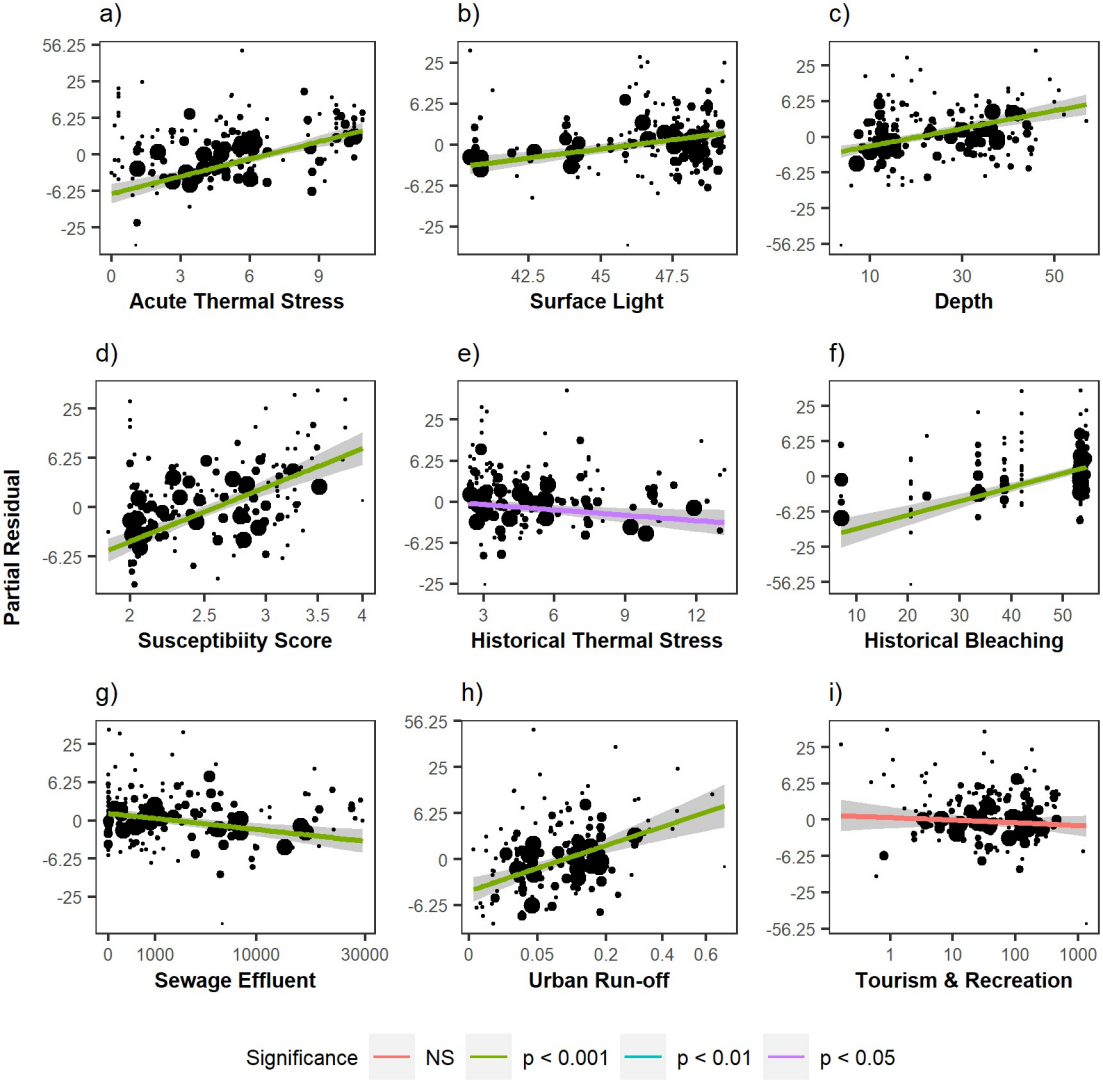
Partial regression plots. Solid line: Back-transformed predicted percent bleached from best-fit model minus the intercept of the reduced model (best-fit model when the variable of interest and all interactions are removed) across range of (a) acute thermal stress (DHW), (b) surface light (PAR), (c) depth (ft), (d) taxonomic susceptibility score, (e) historical thermal stress (DHW), (f) historic percent bleached (%), (g) sewage effluent, (h) urban run-off, and (i) tourism and recreation. Grey area: upper and lower 95% confidence intervals. Points are the back-transformed residuals of the reduced fit model. Residuals are sized by observation weight. See S8 Table for variable descriptions.

From largest to smallest effect size relative to other predictors, historical bleaching, taxonomic susceptibility, acute thermal stress, urban run-off, depth, and PAR were positively correlated with percent bleached in 2019, while sewage effluent and historical thermal stress had a negative relationship with percent bleached (Table 1, Fig 6, S8 Fig). Acute thermal stress interacted significantly with historical thermal stress and tourism (Table 1, S7 Table, S8 Fig). Taxonomic susceptibility interacted significantly with urban run-off, historical bleaching, and depth. Together, these terms and interactions accounted for 76% of the variation in bleaching (S6 Fig).

**Table 1:** Best-fit model for drivers of 2019 bleaching (adjusted R-squared = 0.76).

Variable	Estimate	SE	t-value	p-value
Historic % Bleached	1.321	0.158	8.385	< 0.0001
Taxonomic Susceptibility Score	1.297	0.166	7.812	< 0.0001
Acute Thermal Stress	1.22	0.156	7.8	< 0.0001
Urban Run-off	0.853	0.165	5.183	< 0.0001
Depth	0.774	0.116	6.689	< 0.0001
Surface Light	0.52	0.096	5.409	< 0.0001
Sewage Effluent	-0.491	0.133	-3.709	< 0.001
Historical Thermal Stress	-0.279	0.119	-2.344	< 0.05
Tourism & Recreation	-0.138	0.166	-0.83	0.408
Acute Thermal Stress x Historical Thermal Stress	1.49	0.233	6.385	< 0.0001
Acute Thermal Stress x Tourism & Recreation	0.739	0.143	5.182	< 0.0001
Taxonomic Susceptibility Score x Urban Run-off	0.622	0.145	4.288	< 0.0001
Taxonomic Susceptibility Score x Historic % Bleached	-0.611	0.159	-3.846	< 0.001
Taxonomic Susceptibility Score x Depth	0.334	0.128	2.618	< 0.01

See S8 Table for driver variable descriptions.

**Fig 7:**
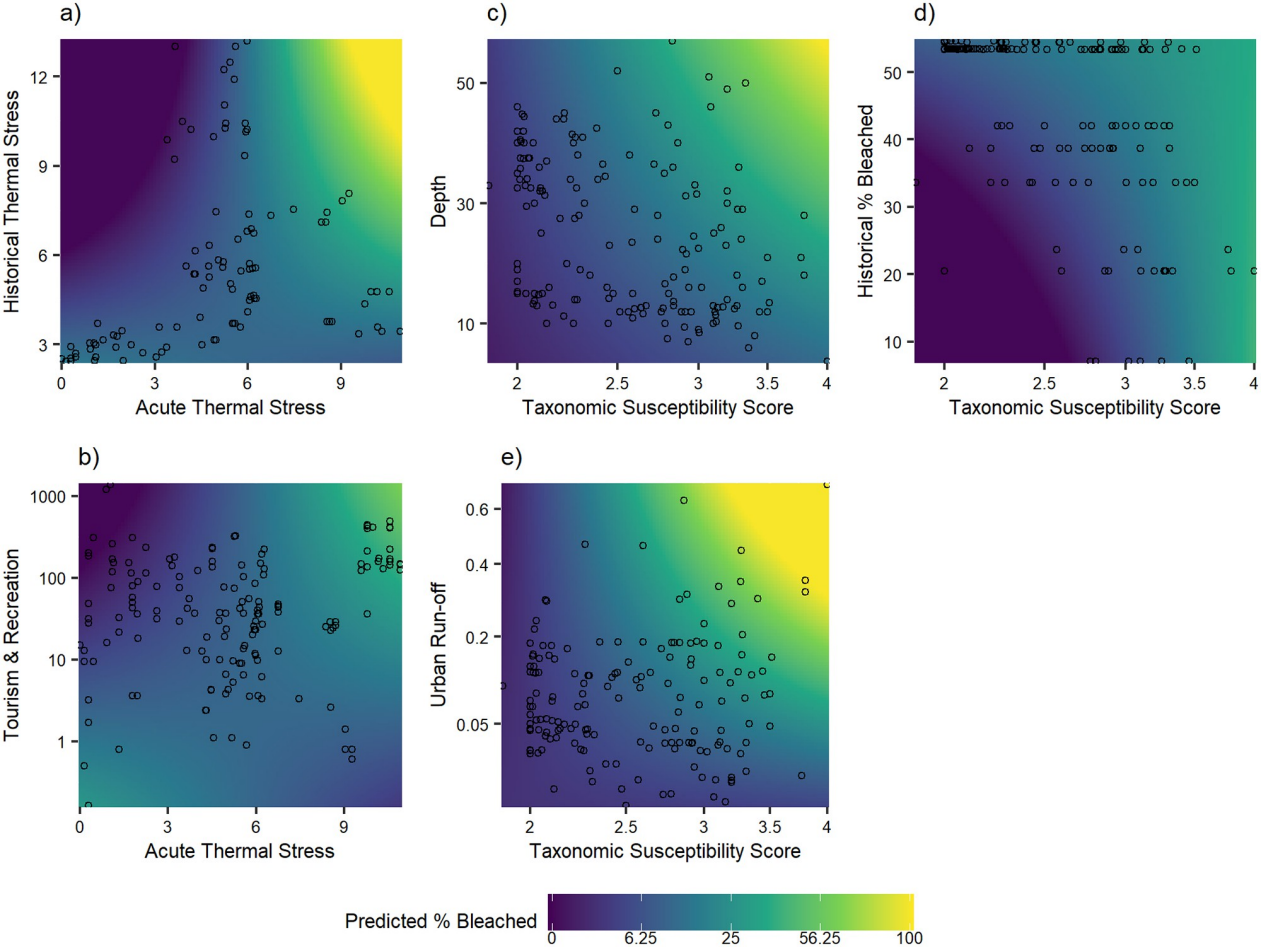
Prediction interaction surface plots. Gradient denotes predicted percent bleached from best-fit model across ranges of (a) acute thermal stress (DHW) and historical thermal stress (DHW), (b) acute thermal stress (DHW) and tourism/recreation, (c) susceptibility and depth (ft), (d) susceptibility and historic percent bleached, and (e) susceptibility and urban run-off. Model predictions were generated per interaction effect by holding all other variables at their mean value. Points denote observed values of variables. See S8 Table for variable descriptions.

When assessing the interaction between acute thermal stress and historical thermal stress, model predictions suggest that percent bleaching peaks at high levels of both acute and historical thermal stress, and is lowest under low acute stress coupled with high historical stress (Fig 7). The interaction between acute thermal stress and tourism exhibited similar patterns, with the highest levels of bleaching predicted under high acute stress and high tourism levels. The interaction between taxonomic susceptibility and depth, historical bleaching, and urban run-off exhibited similar overall trends, with high bleaching extent predicted only under conditions of high taxonomic susceptibility. Even when subjected to high levels of urban run-off, moderate bleaching extent only occurred when susceptibility was above 2.5. In general, predicted bleaching tended to increase with depth, but this pattern was most evident for susceptibility of 3 and above. Across the range of historical bleaching observed, predicted levels of bleaching were similar when susceptibility reached 3.5. With lower susceptibility, predicted bleaching tended to increase with increased historical bleaching.

When the best-fit model was perturbed by increasing and decreasing each driver variable in turn by 1 SD (holding all other drivers constant at their mean values), the largest increase in mean predicted bleaching per driver was due to increasing taxonomic susceptibility, with bleaching increasing from 15.6% to 26.1% (S9 Fig). This was closely followed by an increase in acute thermal stress (24.7%) and urban run-off (23.6%). Reducing historical bleaching (%) resulted in the largest decline in predicted bleaching (15.6% to 7.03%), followed by acute thermal stress (15.6% to 7.29%) and taxonomic susceptibility (15.6% to 8.13%).

**Fig 8:**
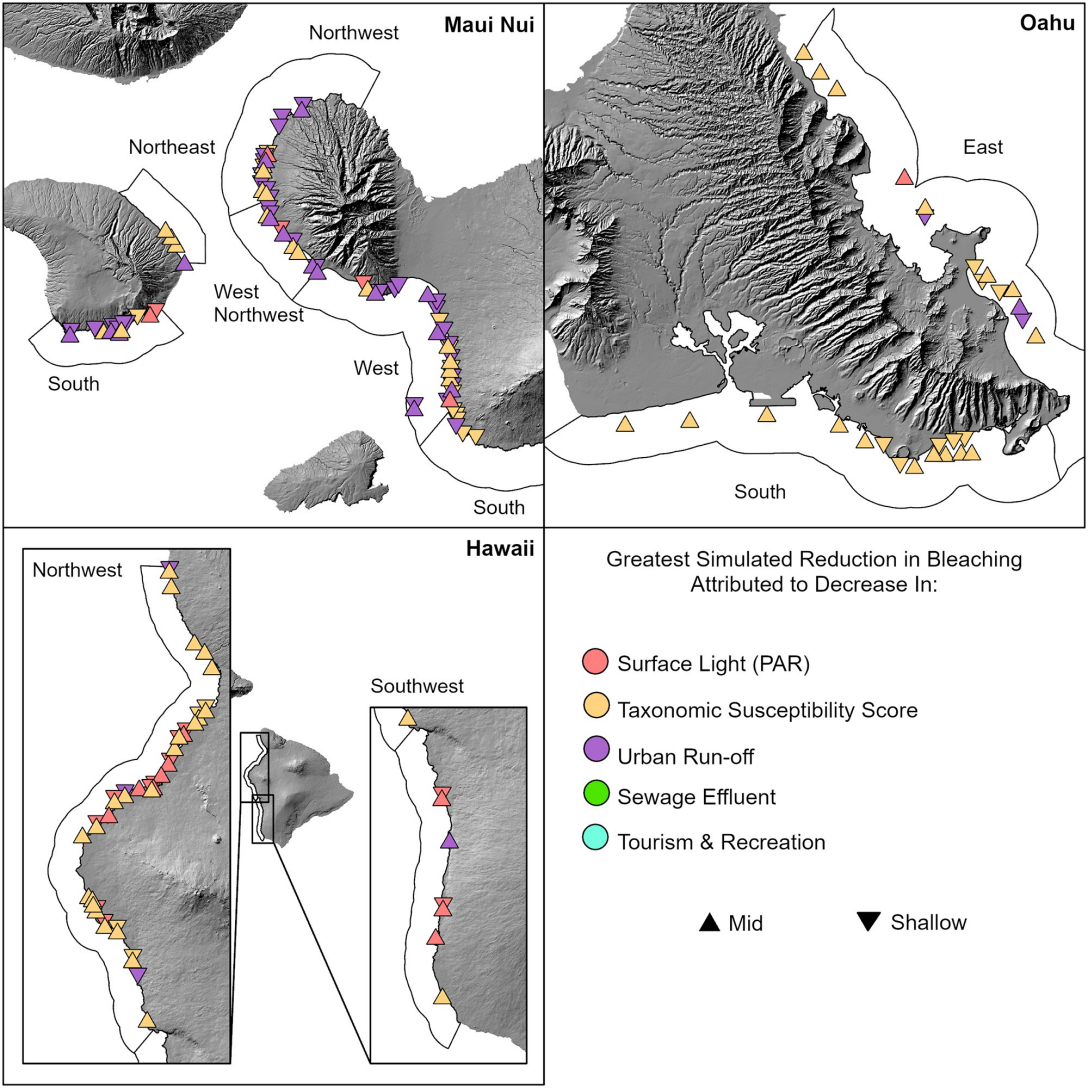
Potential site-specific management actions to reduce bleaching under simulated thermal stress using best-fit model. Colors reflect the variable found to result in the largest decrease in predicted bleaching (%) at the site-level under model perturbations, wherein all variables were in turn reduced by 1 SD as other variables were held at observed values. Only variables that could feasibly be managed were perturbed (surface light (PAR), sewage effluent, urban run-off, susceptibility, and tourism/recreation). See S8 Table for driver variable descriptions.

Management actions that would best serve to mitigate the effects of future thermal stress events proved to be highly variable across sites (Fig 8). However, most sites in Maui and Lānaʻi appeared to benefit from decreasing urban run-off under simulated thermal stress, while on O‘ahu bleaching would decrease most following a decline in taxonomic susceptibility. In west Hawai‘i Island, decreasing surface light (PAR) as well as taxonomic susceptibility emerged as recommended management interventions. No clear patterns between depths emerged.

## Discussion

### Coral bleaching in 2019 was driven by community composition & environmental factors

A gradient of bleaching responses was observed across the MHI during the 2019 event, which contrasted patterns in 2015 when thermal stress was more extreme and more uniform mass bleaching was observed. This variability in bleaching was found to be largely driven by taxonomic susceptibility, consistent with prior studies illustrating the significant impact that the distribution of vulnerable taxa has on the outcome of a bleaching event [39, 55, 56]. The 2019 surveys showed that the coral community assemblage on O‘ahu was relatively more susceptible to bleaching than other islands. On O‘ahu, the most vulnerable taxa may have survived the 2015 event due to the lower thermal stress (0.5°C-weeks) relative to other islands. While lower bleaching and thermal stress was observed on Hawai‘i Island in 2019 compared to 2015, it is important to note that Hawai‘i Island experienced a catastrophic loss of coral cover from the 2015 bleaching event, with mortality recorded in more than half of all stony corals present on the island’s west coast [38]. The loss of corals likely included taxa more susceptible to bleaching, such as *P*. *meandrina −* colonies that averaged 77.6% total post-bleaching mortality following the 2015 event. The resulting communities, therefore, were likely less susceptible overall and when they were exposed to lower acute thermal stress in 2019, they bleached less. While massive *Porites* colonies are considered stress tolerant in other contexts [28], certain communities on Hawai‘i Island’s west coast suffered significant post-bleaching mortality of *P*. *lobata* and *P*. *evermanni* in 2015 (losses of 55.7% and 92.5%, respectively [38]). In 2019, the bleaching responses of *P*. *lobata* and *P*. *lutea* were amongst the mildest at each island surveyed. This shift in bleaching response is likely more related to the lower DHW experienced in 2019 vs. 2015, rather than changes in susceptibility operating at the colony-level. While it is hypothesized that coral populations may adapt and/or acclimate to thermal stress [23, 29, 36], we lack the region-wide historical bleaching data that would provide robust support for taxa-specific changes in thermal tolerance.

Regional and site-level bleaching may also be strongly driven by physiological variation of the host and symbiont. Fluorescent tissue pigment granules (FPG) are known to protect corals from broad-spectrum solar radiation [57], and fast-growing pocilloporids have been reported to have lower densities compared to slow-growing massive poritids. In addition, thinner tissues in pocilloporids compared to poritids could make them more vulnerable to thermal stress [24]. More so, differences in the dominant symbiont genera appear to drive intraspecific variability in colony-level thermal tolerance [58–60]. For example, the bleaching response of *Montipora capitata* in Kāne‘ohe Bay during the 2015 bleaching event was significantly driven by dominant symbiont genus, with some colonies severely bleaching while others remained unaffected [60]. Beyond local abiotic conditions, differences in symbionts, together with host genotypic factors, may explain the observed species-level bleaching variability across islands; however, further research is warranted.

Although taxonomic susceptibility was the strongest predictor of the 2019 bleaching, thermal stress was a major driver and often played a strong interactive role with other natural and anthropogenic factors. These results are consistent with the bleaching response to thermal stress documented in 2014/2015 [39, 40]. The 2019 response was mediated by both historical and acute thermal stress. Importantly, reefs were predicted to bleach more if they experienced both high acute thermal stress and high historical thermal stress, but predicted to bleach less if they experienced high acute thermal stress and low historical thermal stress. These results suggest that previous exposure to bleaching did not result in significant overall acclimation or adaptation to thermal stress events at the reef community level. Moreover, the significant interaction between historical bleaching and susceptibility demonstrated that corals with high susceptibility to bleaching will bleach regardless of their previous bleaching response. Surprisingly, SST variability did not emerge as a significant driver of bleaching in 2019. This is also contradictory to findings in other regions, where coral bleaching is significantly less common in localities with high SST variability [15, 23, 61, 62].

Other environmental drivers of the 2019 bleaching event included surface light (PAR). This was a positive driver of bleaching during 2019 according to this study and has frequently been implicated in increasing bleaching during periods of elevated thermal stress by amplifying photo inhibition [19, 63, 64]. Light attenuation, or turbidity, can also regulate bleaching by reducing severe irradiance; corals at locations with high turbidity or cloud cover have been found to bleach less [37, 64, 65]. However, turbidity modeled using satellite-derived kdPAR measurements in this study did not emerge as a significant mediator of the 2019 bleaching.

The role of depth in bleaching patterns varies considerably across studies. Numerous studies have found decreased bleaching and bleaching-induced mortality with depth, but this effect often varies due to complex and interacting local factors such as coral community composition, temperature, and light attenuation [66, 67]. While Couch et al. [39] found a slight negative relationship between depth and bleaching during the 2014 bleaching event in the NWHI, the 2019 bleaching response in the MHI tended to increase with depth. The modeled significant interaction between susceptibility and depth predicted that the increase in bleaching at depth was more pronounced for corals with higher susceptibility. Our findings are in line with Venegas et al. [68], who found no meaningful depth refuge from thermal stress down to 38 m in the west and central Pacific Ocean. Also, bleaching thresholds can vary with depth, with higher sensitivities in deeper waters [62, 69]. Therefore, in the MHI, taxa more susceptible to bleaching may be found on deeper reefs. Given that the depth gradient in this study did not surpass 17 m and the relatively high water clarity at depth in the MHI, it is unsurprising that we found no evidence of depth as a refuge from bleaching.

### Anthropogenic stressors exacerbated the 2019 bleaching response

Several anthropogenic stressors were correlated with the 2019 bleaching response. Urban run-off, which in this study consisted of a proxy for pollutants such as trash, household chemicals, pharmaceuticals, and oil [70], was a positive driver of the 2019 bleaching; this relationship was especially detrimental for more susceptible coral taxa. High incidences of coral disease and mortality have been observed on reefs located near areas of run-off [71]. Many chemicals are detrimental to coral health, with oil being particularly lethal [72]. The effects of toxic substances may be enhanced at higher temperatures [73]; during a bleaching event, the rapid degradation of pollutants likely amplifies the negative effects of thermal stress on corals.

Tourism and recreation alone did not significantly predict bleaching, but the significant interaction between tourism/recreation and acute thermal stress suggests that the effects of increased tourism/recreation are exacerbated under periods of high thermal stress. In other words, reefs that are exposed to tourism and recreation may bleach more than undisturbed reefs during a bleaching event. The negative impacts of heavy tourism on coral reefs have resulted in higher incidences of damage and disease coupled with lower coral cover observed globally [74, 75].

Sewage effluent was negatively correlated with bleaching. However, the input of sewage into coral reef systems has been historically implicated in elevated levels of disease and macroalgae cover, as well as reduced coral cover, growth, and recruitment [76–78]. While the sewage effluent data used in this study is the best available statewide description of nearshore effluent calculated from estimated total nitrogen and phosphorus flux coming from onsite sewage disposal systems, this data fails to include effluent coming from injection wells. An additional caveat to using this effluent data is that the temporal resolution of the data layer notably predates the 2019 bleaching surveys and thus does not include any additional development nor updates to household cesspools following the generation of this data. Yet the negative relationship between bleaching and effluent as suggested by our best-fit model may support an alternative view of ecosystem resilience described by Côté and Darling [79] in which a degraded ecosystem state increases the abundance of disturbance-tolerant species within a community and boosts the ability of the ecosystem to resist impacts of that disturbance. In this case, we hypothesize that coral communities that persisted in 2019 were able to tolerate high levels of sewage effluent coupled with high thermal stress during the 2015 bleaching event—a sign of positive co-tolerance [80].

### Potential management strategies to mitigate impacts of future thermal stress events

In light of the forecasted increase in bleaching events [81], generating adaptive strategies to mitigate the impacts of these future stress events is essential for coral reef conservation and management. When we simulated a high thermal stress event and perturbed a subset of drivers (that could potentially be manipulated by managers), the impact on predicted bleaching varied substantially across space. This suggests that the effectiveness of these actions may be highly site-specific, which should be interpreted in the context of the operability of these management practices being highly variable across spatial scales [82].

In Maui and Lānaʻi, reducing urban run-off (modelled by calculating area of impervious surface per watershed as a proxy for trash, household chemicals, oil, etc.) appears to have the greatest positive impact on reefs, particularly along the west Maui coastline. The west Maui shoreline is densely populated with resorts, commercial development, and golf courses, with known land-based sources of pollution (LBSP). Long-term monitoring of coral reefs along this coastline has revealed a decline up to 50% in certain impacted areas [83]. The reef off Kahekili Beach Park has been particularly affected by LBSP, with multiple studies linking the loss of coral cover and high proliferation of macro and turf algae to a prevailing nutrient imbalance caused by an influx of nutrient-rich wastewater and chemical toxins from the nearby Lāhainā Wastewater Reclamation Facility [84, 85].

On O‘ahu, our scenarios suggest that a shift to communities composed of less susceptible coral taxa would play a role in the outcomes of future thermal stress events. Managers should be cognizant of how the dominance of stress-tolerant taxa will effect future restoration efforts, and select coral taxa accordingly. However, a narrow focus on bleaching resistance as a primary target for restoration may lead to less diverse reefs if only a subset of resilient taxa are selected. Data from 2019 surveys point to those taxa that appear less susceptible to bleaching; however, it is important to note that the susceptibility of taxa can change over time. Previous studies have demonstrated that under annual bleaching, the susceptibility of certain taxa can reverse by “turning previous ‘winners’ into ‘losers’” [31]. As bleaching events in the Hawaiian Archipelago increase in frequency, we may witness further shifts in taxonomic susceptibility and community assemblages thereafter.

Along west Hawai’i Island, decreasing surface light (PAR) was the most beneficial management action for reducing predicted bleaching at the majority of sites. Shading has been shown to be a direct and effective means of protection against harmful solar radiation for corals [86, 87]. West and Salm [88] recommended that managers consider this proactive measure in response to future forecasted thermal stress events to mitigate bleaching; however, effectively scaling this effort up to the reef-scale is not yet possible, so these results may have limited applicability for meaningful management of future reef-scale resilience.

### Rising frequency of bleaching events and changing trends through time

While ocean temperature trends have indicated an increase in warming over time [3, 89], the 2019 heating event resulted in lower accumulated thermal stress than initially anticipated in the MHI. Thermal stress experienced across the main Hawaiian Islands during the 2019 bleaching event was more moderate than that endured by reefs during the catastrophic 2014/2015 event, and bleaching was either similar or significantly reduced in 2019 compared to 2015. Regardless, marine heatwaves are becoming more frequent and severe across the Hawaiian Archipelago since the first reported bleaching in 1996 (Fig 1, [39]). In the NWHI, the level of thermal stress sustained in 2019 was higher or similar to conditions during previous years, consistent with the warming trend found by Couch et al. [39] and indicating that repeated thermal stress may start extending across more than just the northern atolls. While NWHI corals continue to bleach during these heatwaves, the lower observed bleaching despite higher thermal stress experienced in the northern atolls (PHR and Midway) may be a sign of acclimation in resilient taxa or due to the elimination of vulnerable individuals that did not survive bleaching events prior to 2019 (this study, [39]).

## Conclusions

The 2019 bleaching event had widespread effects on coral reefs across the Hawaiian Archipelago and underscored the rising frequency of thermal stress events not only in the central Pacific, but also around the world. Following the third mass global bleaching event (2014–2017), which resulted in severe mortality, bleaching events have continued to affect reefs globally over the past 5 years including regions such as French Polynesia [90], Bonaire [91], and the Great Barrier Reef [92]. While the 2019 bleaching event in the Hawaiian Islands was not as severe as initially forecasted, future marine heatwaves still harbor the potential for catastrophic impacts. This study highlights the value of large, multi-institutional partnerships to study patterns and processes at spatial scales beyond the scope of any one agency. While the bleaching response was less severe overall across the archipelago in 2019 than in 2014/2015, it was highly variable among sites and taxa—driven largely by the taxonomic susceptibility of the coral assemblages present. Whether the coral communities archipelago-wide exhibited signs of acclimatization to thermal stress is challenging to elucidate, given that potential resilience observed at certain reefs may be directly caused by the massive mortality that followed the 2014/2015 bleaching event and left only the least susceptible taxa present. Further studies should examine changes in coral cover and community composition over time, with an emphasis on collecting taxa-specific size-structure, bleaching and mortality data across a full depth gradient. In light of the forecasted increase in severity and frequency of bleaching events, this work lays important groundwork for predicting the effects of bleaching across space and taxa, and suggests viable management strategies in Hawai‘i for further consideration.

## Supporting information

S1 Table Bleaching survey effort across the Hawaiian Archipelago from August 20 to December 7, 2019, by depth bin (shallow [0–6 m], mid [>6–18 m], deep [>18–30 m]). (DOCX)

S2 Table Overview of survey types used by the Hawaii Coral Bleaching Collaborative to conduct bleaching response surveys in 2019. See [45] for additional organization-specific survey metadata.
(DOCX)

S3 Table Bleaching susceptibility scores across species and genera. All coral taxa were scored from least (1) to most susceptible (5) to bleaching.
(DOCX)

S4 Table Sample size of surveys used to examine trends in taxa-level coral bleaching. (DOCX)

S5 Table Sample size of clusters used in 2019 spatial analysis of bleaching. Blanks indicate depth bins per location where no surveys were conducted, or where clusters were excluded due to low sample sizes.
(DOCX)

S6 Table Sample size of clusters by year used in temporal analysis of bleaching. Only depth bins and zones (MHI) or islands (NWHI) visited in both 2014/15 and 2019, and met the minimum sample size, were retained for analysis.
(DOCX)

S7 Table Sample size of clusters used in drivers of bleaching analysis. Blanks indicate depth bins per location where no surveys were conducted, or where clusters were excluded due to low sample sizes.
(DOCX)

S8 Table Full description of the 13 variables investigated in drivers of 2019 bleaching analysis. Satellite and model-derived environmental data was matched to the bleaching survey data using the mean latitude and longitude per cluster. To handle NA values, a function was used for each driver variable to find the nearest non-NA data pixel using a defined, expanding search radius. All pixels within the distance that the first non-NA pixel was found in were used to calculate the mean of that variable per survey cluster. The maximum search radius defined for all variables was 8 km, except for wave action, which was limited to 750 m.
(DOCX)

S9 Table Variables and results for drivers of 2019 bleaching model selection. See S8 Table for variable descriptions.
(DOCX)

S10 Table Linear mixed model output (LMM) of percent bleached by year (2015 and 2019) and zone. (DOCX)

S1 Fig Bleaching survey effort (# surveys/day) and NOAA Coral Reef Watch’s Degree Heating Weeks (DHW) data for the Northwestern Hawaiian Islands (French Frigate Shoals, Kure Atoll, Lisianski Island, and Pearl and Hermes Atoll) during 2019. (DOCX)

S2 Fig Bleaching survey effort (# surveys/day) and NOAA Coral Reef Watch’s Degree Heating Weeks (DHW) data (bottom of panel) Main Hawaiian Islands (Kaua‘i, O‘ahu, Moloka‘i, Maui, Lānaʻi, and Hawai‘i Island) during 2019. (DOCX)

S3 Fig Box plots of cluster-level observed percent bleached per island (NWHI) or zone (MHI). Points are sized by weights assigned per cluster. Zones are abbreviated in grey along the x-axis.
(DOCX)

S4 Fig Mean percent bleached per cluster across the Northwestern Hawaiian Islands in 2019. Points represent individual clusters, with shape indicating depth bin. Source: Esri, Earthstar Geographics (TerraColor NextGen) imagery.
(DOCX)

S5 Fig Mean percent bleached per cluster across the main Hawaiian Islands in 2019. Points represent individual clusters, with shape indicating depth bin. Zones are denoted by white polygons.
(DOCX)

S6 Fig Box plots of cluster-level percent bleached (%) per island during the 2014 (NWHI) or 2015 (MHI) bleaching event and the 2019 bleaching event (both regions). Points are sized by weights assigned per cluster.
(DOCX)

S7 Fig Box plots of cluster-level percent bleached per zone in the MHI during the 2015 and 2019 bleaching events. Points are sized by weights assigned per cluster.
(DOCX)

S8 Fig Parameter estimates of best-fit model (± SE). See S8 Table for variable descriptions.
(DOCX)

S9 Fig Model perturbation plots. Points represent the mean predicted bleaching (%) determined per model perturbation, when each variable was increased/decreased by 1 SD, with all other variables held at original observed values. Error bars represent standard error of the mean. Black line denotes mean of predicted % bleached by unperturbed model. See S8 Table for variable descriptions.
(DOCX)
