# Supplementary material for: Coral taxonomy and local stressors drive bleaching prevalence across the Hawaiian Archipelago in 2019
Source: PLoS One. 2022 Sep 1;17(9):e0269068. doi: 10.1371/journal.pone.0269068 (PMC9436070; doi:10.1371/journal.pone.0269068)
Supplement: S1 Table — (DOCX) [file pone.0269068.s001.docx]

**S1 Table. Bleaching survey effort across the Hawaiian Archipelago from August 20 to December 7, 2019, by depth bin (shallow [0–6 m], mid [>6–18 m], deep [>18–30 m]).**

| **Region** | **Island** | **n (# surveys)** | | |
| --- | --- | --- | --- | --- |
|  |  | **Shallow** | **Mid** | **Deep** |
| NWHI | Kure | 4 | 9 | 4 |
|  | Pearl and Hermes | 4 | 4 | 2 |
|  | Lisianski | 5 | 4 | 3 |
|  | French Frigate Shoals | 4 | 3 | 0 |
| MHI | Kaua‘i | 200 | 70 | 0 |
|  | O‘ahu | 913 | 69 | 16 |
|  | Moloka‘i | 29 | 0 | 0 |
|  | Lānaʻi | 30 | 24 | 2 |
|  | Maui | 227 | 244 | 2 |
|  | Hawai‘i | 99 | 204 | 2 |
