# Supplementary material for: Coral taxonomy and local stressors drive bleaching prevalence across the Hawaiian Archipelago in 2019
Source: PLoS One. 2022 Sep 1;17(9):e0269068. doi: 10.1371/journal.pone.0269068 (PMC9436070; doi:10.1371/journal.pone.0269068)
Supplement: S2 Table — See [45] for additional organization-specific survey metadata. (DOCX) [file pone.0269068.s002.docx]

**S2 Table. Overview of survey types used by the Hawaii Coral Bleaching Collaborative to conduct bleaching response surveys in 2019. See [43] for additional organization-specific survey metadata.**

| **Survey Type** | **Method Overview** | **Area Surveyed (m^2^)** |
| --- | --- | --- |
| Rapid Visual Assessments | Divers visually assessed the benthic community and structure within a specified survey area and recorded percent live coral cover and percent of coral cover that was bleached. If possible, dominant coral taxa and associated bleaching prevalence were also recorded. | 78.5−393 |
| Transect-Intercept | Divers recorded benthic composition every 0.25 m for the entire transect to species level for living taxa and recorded all non-living substrate. Surveyors assessed and recorded corals as bleached and non-bleached. | 25 |
| Photoquadrat Analysis | Divers collected imagery at 1-m intervals along each transect, and the imagery was analyzed using point count identification to determine the percent cover of each benthic functional group, as well as percent of coral cover that was bleached. | 25 |
