# Supplementary material for: Coral taxonomy and local stressors drive bleaching prevalence across the Hawaiian Archipelago in 2019
Source: PLoS One. 2022 Sep 1;17(9):e0269068. doi: 10.1371/journal.pone.0269068 (PMC9436070; doi:10.1371/journal.pone.0269068)
Supplement: S3 Table — All coral taxa were scored from least (1) to most susceptible (5) to bleaching. (DOCX) [file pone.0269068.s003.docx]

**S3 Table. Bleaching susceptibility scores across species and genera. All coral taxa were scored from least (1) to most susceptible (5) to bleaching.**

| **Taxa** | **Code** | **Susceptibility Score** |
| --- | --- | --- |
| *Acropora gemmifera* | AGEM | 5 |
| *Montipora dilatata* | MDIL | 5 |
| *Pocillopora damicornis* | PDAM | 5 |
| *Pavona duerdeni* | PDUE | 5 |
| *Porites duerdeni* | PODU | 5 |
| *Montipora capitata* | MCAP | 4 |
| *Montipora flabellata* | MFLA | 4 |
| *Montipora incrassata* | MINC | 4 |
| *Pavona maldivensis* | PMAL | 4 |
| *Pocillopora meandrina* | PMEA | 4 |
| *Montipora sp* | MOSP | 3 |
| *Pocillopora grandis* | PGRA | 3 |
| *Porites lichen* | PLIC | 3 |
| *Porites lutea* | PLUT | 3 |
| *Porites monticulosa* | PMON | 3 |
| *Leptastrea transversa* | LTRA | 2 |
| *Montipora patula* | MPAT | 2 |
| *Porites brighami* | PBRI | 2 |
| *Porites compressa* | PCOM | 2 |
| *Porites lobata* | PLOB | 2 |
| *Porites sp* | POSP | 2 |
| *Pavona varians* | PVAR | 2 |
| *Cycloseris vaughani* | CVAU | 1 |
| *Fungia scutaria* | FSCU | 1 |
| *Leptastrea sp* | LEPT | 1 |
| *Leptoseris incrustans* | LINC | 1 |
| *Leptastrea purpurea* | LPUR | 1 |
| *Psammocora nierstraszi* | PNIE | 1 |
| *Porites rus* | PRUS | 1 |
