# Supplementary material for: Coral taxonomy and local stressors drive bleaching prevalence across the Hawaiian Archipelago in 2019
Source: PLoS One. 2022 Sep 1;17(9):e0269068. doi: 10.1371/journal.pone.0269068 (PMC9436070; doi:10.1371/journal.pone.0269068)
Supplement: S4 Table — (DOCX) [file pone.0269068.s004.docx]

**S4 Table. Sample size of surveys used to examine trends in taxa-level coral bleaching.**

| **Island** | **n (# surveys)** | | |
| --- | --- | --- | --- |
|  | **Shallow** | **Mid** | **Deep** |
| O‘ahu | 19 | 29 | 16 |
| Lānaʻi | 5 | 9 | 2 |
| Maui | 38 | 49 | 2 |
| Hawai‘i | 99 | 204 | 2 |
