# Supplementary material for: Coral taxonomy and local stressors drive bleaching prevalence across the Hawaiian Archipelago in 2019
Source: PLoS One. 2022 Sep 1;17(9):e0269068. doi: 10.1371/journal.pone.0269068 (PMC9436070; doi:10.1371/journal.pone.0269068)
Supplement: S5 Table — Blanks indicate depth bins per location where no surveys were conducted, or where clusters were excluded due to low sample sizes. (DOCX) [file pone.0269068.s005.docx]

**S5 Table. Sample size of clusters used in 2019 spatial analysis of bleaching.** Blanks indicate depth bins per location where no surveys were conducted, or where clusters were excluded due to low sample sizes.

| **Region** | **Island** | **Zone** | **n (# clusters)** | | |
| --- | --- | --- | --- | --- | --- |
|  |  |  | **Shallow** | **Mid** | **Deep** |
| NWHI | Kure | | 4 | 7 | 4 |
|  | Pearl and Hermes | | 3 | 4 |  |
|  | Lisianski | | 5 | 4 | 3 |
|  | French Frigate Shoals | | 3 |  |  |
| MHI | Kaua‘i | North | 4 |  |  |
|  |  | South | 3 |  |  |
|  | O‘ahu | North | 13 | 7 | 6 |
|  |  | East | 33 | 11 | 7 |
|  |  | South | 11 | 11 |  |
|  |  | West | 12 |  |  |
|  | Moloka‘i | Southeast | 3 |  |  |
|  |  | South | 6 |  |  |
|  | Lānaʻi | Northeast |  | 4 |  |
|  |  | South | 8 | 6 |  |
|  | Maui | North | 10 | 9 |  |
|  |  | Southeast | 5 | 7 |  |
|  |  | South | 6 |  |  |
|  |  | West | 25 | 16 |  |
|  |  | West Northwest | 7 | 8 |  |
|  |  | Northwest | 9 | 6 |  |
|  | Hawai‘i | East | 3 | 3 |  |
|  |  | Southwest | 5 | 9 |  |
|  |  | Northwest | 19 | 32 |  |
