# Supplementary material for: Coral taxonomy and local stressors drive bleaching prevalence across the Hawaiian Archipelago in 2019
Source: PLoS One. 2022 Sep 1;17(9):e0269068. doi: 10.1371/journal.pone.0269068 (PMC9436070; doi:10.1371/journal.pone.0269068)
Supplement: S6 Table — Only depth bins and zones (MHI) or islands (NWHI) visited in both 2014/15 and 2019, and met the minimum sample size, were retained for analysis. (DOCX) [file pone.0269068.s006.docx]

**S6 Table. Sample size of clusters by year used in temporal analysis of bleaching.** Only depth bins and zones (MHI) or islands (NWHI) visited in both 2014/15 and 2019, and met the minimum sample size, were retained for analysis.

| **Region** | **Island** | **Zone** | **n (# clusters)** | | | | |
| --- | --- | --- | --- | --- | --- | --- | --- |
|  |  |  | **Shallow** | | **Mid** | | |
|  |  |  | **2015** | **2019** | **2014** | **2015** | **2019** |
| NWHI | Pearl and Hermes | |  |  | 5 |  | 4 |
|  | Lisianski | |  |  | 7 |  | 4 |
| MHI | O‘ahu | East | 15 | 33 |  |  |  |
|  |  | Southwest | 12 | 11 |  | 8 | 11 |
|  | Lānaʻi | Northeast |  |  |  | 8 | 4 |
|  |  | Southwest |  |  |  | 16 | 6 |
|  | Maui | West | 9 | 25 |  | 9 | 16 |
|  |  | West Northwest |  |  |  | 3 | 8 |
|  |  | Northwest | 4 | 9 |  | 5 | 6 |
|  | Hawai‘i | Southwest |  |  |  | 3 | 9 |
|  |  | Northwest | 3 | 19 |  | 49 | 32 |
