# Supplementary material for: Coral taxonomy and local stressors drive bleaching prevalence across the Hawaiian Archipelago in 2019
Source: PLoS One. 2022 Sep 1;17(9):e0269068. doi: 10.1371/journal.pone.0269068 (PMC9436070; doi:10.1371/journal.pone.0269068)
Supplement: S7 Table — Blanks indicate depth bins per location where no surveys were conducted, or where clusters were excluded due to low sample sizes. (DOCX) [file pone.0269068.s007.docx]

**S7 Table. Sample size of clusters used in drivers of bleaching analysis.** Blanks indicate depth bins per location where no surveys were conducted, or where clusters were excluded due to low sample sizes.

| **Island** | **Zone** | **n (# clusters)** | |
| --- | --- | --- | --- |
|  |  | **Shallow** | **Mid** |
| Hawai‘i | Southwest | 2 | 5 |
|  | Northwest | 16 | 29 |
| Lānaʻi | Northeast |  | 4 |
|  | Southwest | 8 | 6 |
| Maui | South | 6 |  |
|  | West | 17 | 11 |
|  | West Northwest | 7 | 8 |
|  | Northwest | 9 | 6 |
| O‘ahu | East | 5 | 9 |
|  | South | 5 | 10 |
