# Supplementary material for: Coral taxonomy and local stressors drive bleaching prevalence across the Hawaiian Archipelago in 2019
Source: PLoS One. 2022 Sep 1;17(9):e0269068. doi: 10.1371/journal.pone.0269068 (PMC9436070; doi:10.1371/journal.pone.0269068)
Supplement: S8 Table — Satellite and model-derived environmental data was matched to the bleaching survey data using the mean latitude and longitude per cluster. To handle NA values, a function was used for each driver variable to find the nearest non-NA data pixel using a defined, expanding search radius. All pixels within the distance that the first non-NA pixel was found in were used to calculate the mean of that variable per survey cluster. The maximum search radius defined for all variables was 8 km, except for wave action, which was limited to 750 m. (DOCX) [file pone.0269068.s008.docx]

**S8** **Table. Full description of the 13 variables investigated in drivers of 2019 bleaching analysis.** Satellite and model-derived environmental data was matched to the bleaching survey data using the mean latitude and longitude per cluster. To handle NA values, a function was used for each driver variable to find the nearest non-NA data pixel using a defined, expanding search radius. All pixels within the distance that the first non-NA pixel was found in were used to calculate the mean of that variable per survey cluster. The maximum search radius defined for all variables was 8 km, except for wave action, which was limited to 750 m.

| **Variable** | **Description** | **Original Source** |
| --- | --- | --- |
| Acute Thermal Stress | Mean of the maximum Degree Heating Week events experienced during the one year period before the survey date (2018-2019) | NOAA Coral Reef Watch. 2018. Updated daily. NOAA Coral Reef Watch Version 3.1 Daily  Global 5km Satellite Coral Bleaching Degree Heating Week Product, Mar. 25, 1985-Dec. 31, 2019. College Park, Maryland, USA: NOAA Coral Reef Watch. Data set accessed 2020-11-02 at https://coastwatch.pfeg.noaa.gov/erddap/griddap/NOAA_DHW.html. |
| Historical Thermal Stress | Mean of the maximum Degree Heating Week events experienced over ten years leading up to 365 days prior to the survey date (2008-2018) | NOAA Coral Reef Watch. 2018. Updated daily. NOAA Coral Reef Watch Version 3.1 Daily  Global 5km Satellite Coral Bleaching Degree Heating Week Product, Mar. 25, 1985-Dec. 31, 2019. College Park, Maryland, USA: NOAA Coral Reef Watch. Data set accessed 2020-11-02 at https://coastwatch.pfeg.noaa.gov/erddap/griddap/NOAA_DHW.html. |
| Sea Surface Temperature Variability | Mean of the weekly range of sea surface temperatures experienced from 1985 to the 2019 survey date | NOAA Coral Reef Watch. 2019. Updated daily. NOAA Coral Reef Watch Version 3.1 Daily  5km Satellite Regional Virtual Station Time Series Data for Hawaii, Mar. 25, 1985-Dec. 31, 2019. College Park, Maryland, USA: NOAA Coral Reef Watch. Data set accessed 2020-11-02 at https://oceanwatch.pifsc.noaa.gov/erddap/griddap/CRW_sst_v1_0.html. |
| Depth | The depth in feet at which bleaching surveys were conducted. | This study |
| Surface Light | Photosynthetically available radiation (PAR) calculated from 8 day measurements collected leading up to the 2019 bleaching event and following the peak (7/24/19−11/21/2019). | NASA Goddard Space Flight Center, Ocean Ecology Laboratory, Ocean Biology Processing  Group. Visible and Infrared Imager/Radiometer Suite (VIIRS) Photosynthetically Available Radiation Data; 2018 Reprocessing. NASA OB.DAAC, Greenbelt, MD, USA. doi: data/10.5067/NPP/VIIRS/L3M/PAR/2018. Accessed on 11/12/2020. |
| Light Attenuation | Calculated from weekly measurements collected leading up the 2019 bleaching event and following the peak (7/23/2019−11/19/2019). | Son, S. and M. Wang. 2015. Diffuse attenuation coefficient of the photosynthetically available  radiation Kd(PAR) for global open ocean and coastal waters. Remote Sensing of Environment 159: 250-258. doi:10.1016/j.rse.2014.12.011, provided by NOAA CoastWatch/OceanWatch. Accessed on 11/12/2020. https://coastwatch.pfeg.noaa.gov/erddap/griddap/nesdisVHNSQkdparWeekly.html |
| Wave Action | Global WaveWatchIII data shadowed by coastlines using Incident Wave Swath (IWS); watts per meter of wave front (W m^-1^), 1979−2012 climatology | Cheung, K.F. 2010. WaveWatch III (WW3) Global Wave Model. 1979-2012. Distributed by the Pacific Islands Ocean Observing System (PacIOOS) http://pacioos.org/metadata/ww3_global.html. Accessed 2016-08-31. |
| Historic % Bleached | Percent of live coral that was bleached observed on surveys conducted during the last major bleaching event (MHI: 2015; NWHI: 2014). In the MHI, zone level means were calculated; in the NWHI, island/atoll level means were calculated due to differences in exact sampling locations between 2014/2015 and 2019. | Couch, C. S., J. H. R. Burns, G. Liu, K. Steward, T. N. Gutlay, J. Kenyon, C. M. Eakin, and R. K. Kosaki. 2017. Mass coral bleaching due to unprecedented marine heatwave in Papahānaumokuākea Marine National Monument (Northwestern Hawaiian Islands). PLoS ONE 12:e0185121.  Hawai‘i Coral Bleaching Collaborative. 2021. Hawai‘i Coral Bleaching Collaborative: surveys for percent of bleached coral cover across the Hawaiian Archipelago from October 02 to November 18, 2015, https://www.fisheries.noaa.gov/inport/item/64755. |
| Taxonomic Susceptibility Score | All taxa were assigned a score from (1) least susceptible to (5) most susceptible to bleaching using a combination of taxa percent bleaching levels and bleaching severity in 2019 (S5 Table), scores developed in previous analyses (Couch et al. 2017), and unpublished data from NOAA PIFSC’s Ecosystem Sciences Division. See Methods & Materials for calculation used to derive community susceptibility per survey and Appendix 1: Table S3 for taxa-specific scores. | This study |
| Sewage Effluent | Total effluent composed of nitrogen and phosphorus flux from onsite waste disposal systems (OSDS) (gal/km^2/d) from 2008 (O‘ahu) and 2010 (Maui, Lānaʻi, Hawai‘i). | Wedding, L.M., J. Lecky, J.M. Gove, H.R. Walecka, M.K. Donovan, G.J. Williams, J.-B.  Jouffray, L.B. Crowder, A. Erickson, K. Falinski, A.M. Friedlander, C.V. Kappel, J.N. Kittinger, K. McCoy, A. Norström, M. Nyström, K.L.L. Oleson, K.A. Stamoulis, C. White, and K.A. Selkoe. 2018 Advancing the integration of spatial data to map human and natural drivers on coral reefs. PLoS ONE 13(3): e0189792 |
| Tourism & Recreation | Direct human impact approximated by calculation of annual average number of photo users per day per location for the years 2005−2014. | Lecky, J. 2016. Ecosystem vulnerability and mapping cumulative impacts on Hawaiian reefs. Thesis. University of Hawai‘i at Mānoa, Honolulu, Hawai‘i, USA. |
| Agricultural Run-off | Proxy for nutrient runoff (fertilizers) and chemical runoff (pesticides and herbicides) produced by calculating the area of agricultural land and golf courses by watershed from 2010/2011. | Lecky, J. 2016. Ecosystem vulnerability and mapping cumulative impacts on Hawaiian reefs. Thesis. University of Hawai‘i at Mānoa, Honolulu, Hawai‘i, USA. |
| Urban Run-off | Proxy for trash, household chemicals, oil, etc. produced by calculating area of impervious surface per watershed from 2010/2011. | Lecky, J. 2016. Ecosystem vulnerability and mapping cumulative impacts on Hawaiian reefs. Thesis. University of Hawai‘i at Mānoa, Honolulu, Hawai‘i, USA. |
