# Supplementary material for: Coral taxonomy and local stressors drive bleaching prevalence across the Hawaiian Archipelago in 2019
Source: PLoS One. 2022 Sep 1;17(9):e0269068. doi: 10.1371/journal.pone.0269068 (PMC9436070; doi:10.1371/journal.pone.0269068)
Supplement: S9 Table — See S8 Table for variable descriptions. (DOCX) [file pone.0269068.s009.docx]

**S9 Table. Variables and results for drivers of 2019 bleaching model selection.** See S8 Table for variable descriptions.

| Variable | Transformation | Best-fit model | Conclusion |
| --- | --- | --- | --- |
| **Acute Thermal Stress** | N/A | **Y** | **Significant** |
| *x Surface Light* |  | N | N/A |
| *x Light Attenuation* |  | N | N/A |
| *x Depth* |  | N | N/A |
| *x Wave Action* |  | N | N/A |
| *x Taxonomic Susceptibility Score* |  | N | N/A |
| ***x Historical Thermal Stress*** | N/A | **Y** | **Significant** |
| *x Historic % Bleached* |  | N | N/A |
| *x Sea Surface Temperature Variability* |  | N | N/A |
| *X Sewage Effluent* |  | N | N/A |
| *x Agricultural Run-off* |  | N | N/A |
| *x Urban Run-off* |  | N | N/A |
| ***x Tourism & Recreation*** |  | **Y** | **Significant** |
| Sea Surface Temperature Variability |  | N | N/A |
| **Taxonomic Susceptibility Score** | log | **Y** | **Significant** |
| *x Surface Light* |  | N | N/A |
| *x Light Attenuation* |  | N | N/A |
| ***x Depth*** |  | **Y** | **Significant** |
| *x Wave Action* |  | N | N/A |
| ***x Historic % Bleached*** |  | **Y** | **Significant** |
| *x Historical Thermal Stress* |  | N | N/A |
| *x Sea Surface Temperature Variability* |  | N | N/A |
| *x Sewage Effluent* |  | N | N/A |
| *x Agricultural Run-off* |  | N | N/A |
| ***x Urban Run-off*** |  | **Y** | **Significant** |
| *x Tourism & Recreation* |  | N | N/A |
| **Surface Light** | N/A | **Y** | **Significant** |
| Light Attenuation | N/A | N | N/A |
| **Depth** | N/A | **Y** | **Significant** |
| Wave Action | square-root | N | N/A |
| **Historical Thermal Stress** | N/A | **Y** | **Significant** |
| **Historic % Bleached** | N/A | **Y** | **Significant** |
| **Sewage Effluent** | square-root | **Y** | **Significant** |
| Agricultural Run-off | square-root | N | N/A |
| **Urban Run-off** | square-root | **Y** | **Significant** |
| **Tourism & Recreation** | log | **Y** | Not significant |
