# Supplementary material for: Coral taxonomy and local stressors drive bleaching prevalence across the Hawaiian Archipelago in 2019
Source: PLoS One. 2022 Sep 1;17(9):e0269068. doi: 10.1371/journal.pone.0269068 (PMC9436070; doi:10.1371/journal.pone.0269068)
Supplement: S10 Table — (DOCX) [file pone.0269068.s010.docx]

**S10 Table. Linear mixed model output (LMM) of percent bleached by year (2015 and 2019) and zone.**

| **Random effects** | **Variance** | **SD** |  |
| --- | --- | --- | --- |
| Island (Intercept) | 0.00587 | 0.07661 |  |
| **Fixed effects** | **Estimate** | **SE** | **t-value** |
| (Intercept) | 7.23E+00 | 1.80E-01 | 6.76E-09 |
| Year2019 | -4.98E+00 | 2.62E-01 | 3.15E+02 |
| ZoneNameHawaii_SW | 5.48E-03 | 7.58E-01 | 3.15E+02 |
| ZoneNameLanai_NE | -2.32E+00 | 6.29E-01 | 2.50E-07 |
| ZoneNameLanai_S | -1.42E+00 | 4.51E-01 | 6.60E-08 |
| ZoneNameMaui_NW | -9.89E-01 | 5.30E-01 | 1.26E-07 |
| ZoneNameMaui_W | 3.49E-02 | 4.16E-01 | 4.77E-08 |
| ZoneNameMaui_WNW | 1.08E-02 | 1.05E+00 | 1.97E-06 |
| ZoneNameOahu_E | -1.12E+00 | 5.91E-01 | 1.95E-07 |
| ZoneNameOahu_S | -2.97E+00 | 3.29E-01 | 1.87E-08 |
| Year2019:ZoneNameHawaii_SW | 2.40E+00 | 1.48E+00 | 3.15E+02 |
| Year2019:ZoneNameLanai_NE | 3.64E+00 | 1.54E+00 | 3.15E+02 |
| Year2019:ZoneNameLanai_S | 2.33E+00 | 1.03E+00 | 3.15E+02 |
| Year2019:ZoneNameMaui_NW | 3.94E+00 | 7.86E-01 | 3.15E+02 |
| Year2019:ZoneNameMaui_W | 1.70E+00 | 5.02E-01 | 3.15E+02 |
| Year2019:ZoneNameMaui_WNW | 3.40E+00 | 1.28E+00 | 3.15E+02 |
| Year2019:ZoneNameOahu_E | 2.88E+00 | 7.38E-01 | 3.15E+02 |
