# Supplementary material for: Coral taxonomy and local stressors drive bleaching prevalence across the Hawaiian Archipelago in 2019
Source: PLoS One. 2022 Sep 1;17(9):e0269068. doi: 10.1371/journal.pone.0269068 (PMC9436070; doi:10.1371/journal.pone.0269068)
Supplement: S1 Fig — (DOCX) [file pone.0269068.s011.docx]

**
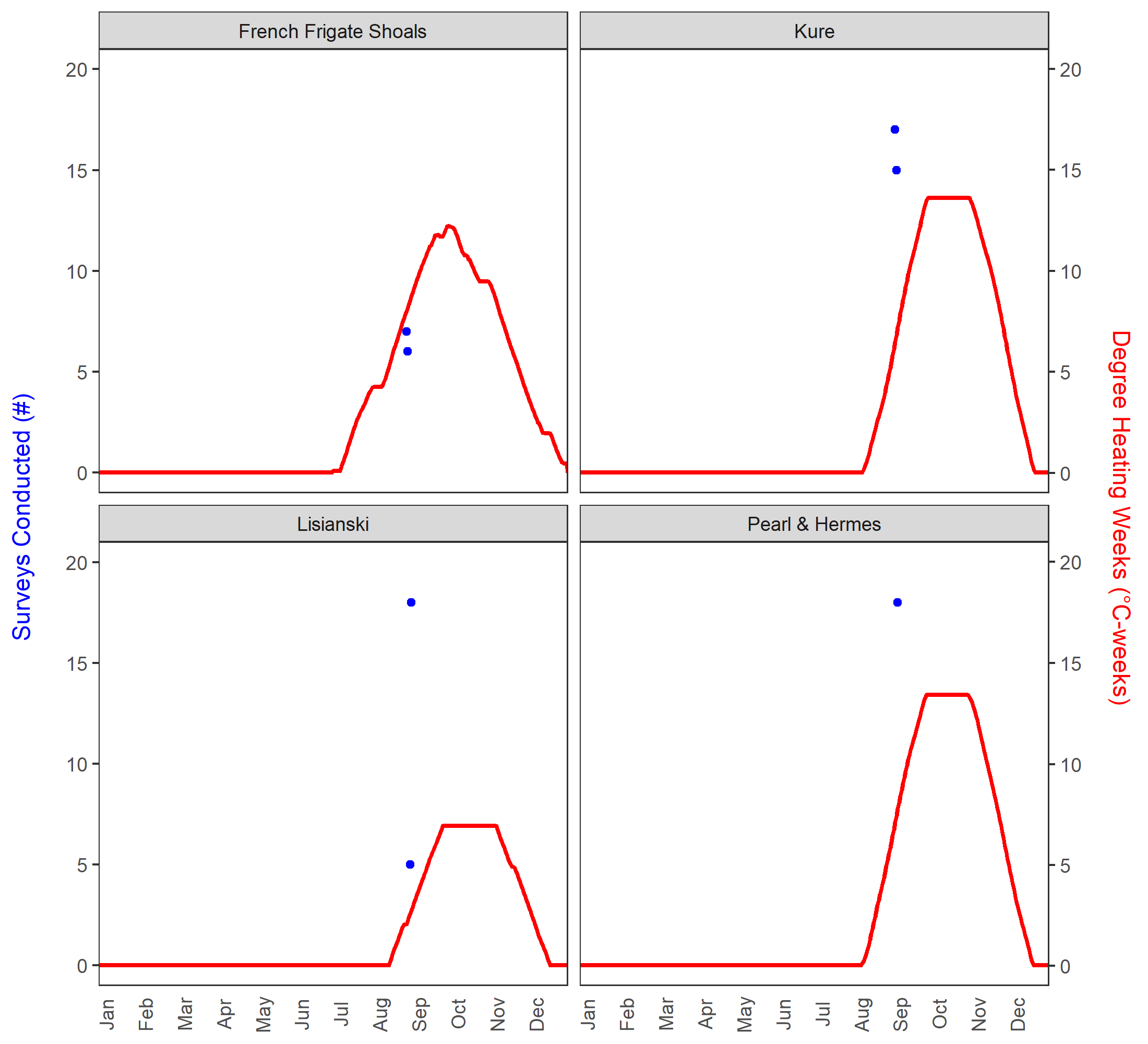
S1 Figure. Bleaching survey effort (# surveys/day) and NOAA Coral Reef Watch’s Degree Heating Weeks (DHW) data for the Northwestern Hawaiian Islands (French Frigate Shoals, Kure Atoll, Lisianski Island, and Pearl and Hermes Atoll) during 2019.**
