# Supplementary material for: Coral taxonomy and local stressors drive bleaching prevalence across the Hawaiian Archipelago in 2019
Source: PLoS One. 2022 Sep 1;17(9):e0269068. doi: 10.1371/journal.pone.0269068 (PMC9436070; doi:10.1371/journal.pone.0269068)
Supplement: S2 Fig — (DOCX) [file pone.0269068.s012.docx]

**
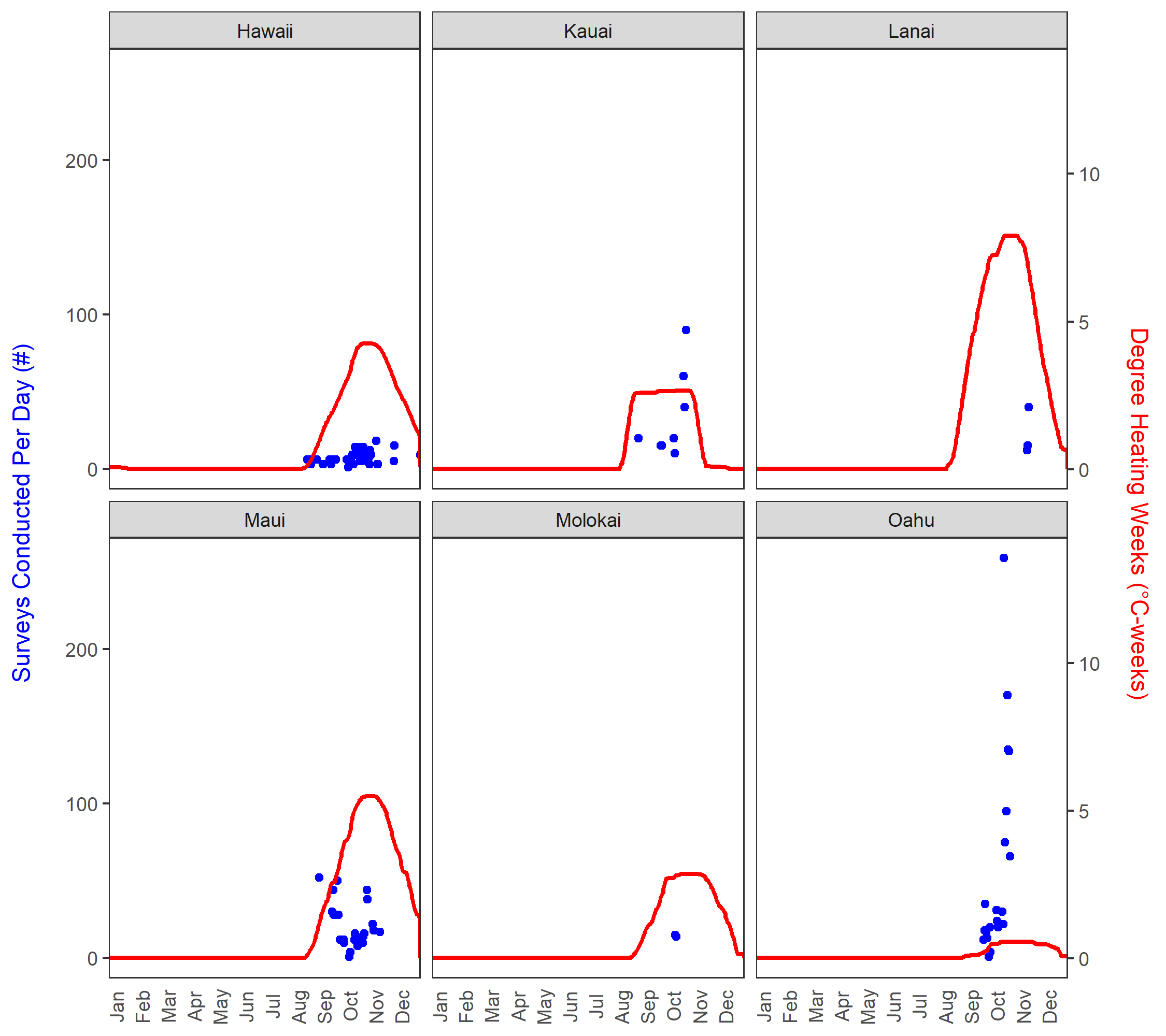
S2 Figure. Bleaching survey effort (# surveys/day) and NOAA Coral Reef Watch’s Degree Heating Weeks (DHW) data (bottom of panel) Main Hawaiian Islands (Kaua‘i, O‘ahu, Moloka‘i, Maui, Lānaʻi, and Hawai‘i Island) during 2019.**
