# Supplementary material for: Coral taxonomy and local stressors drive bleaching prevalence across the Hawaiian Archipelago in 2019
Source: PLoS One. 2022 Sep 1;17(9):e0269068. doi: 10.1371/journal.pone.0269068 (PMC9436070; doi:10.1371/journal.pone.0269068)
Supplement: S3 Fig — Points are sized by weights assigned per cluster. Zones are abbreviated in grey along the x-axis. (DOCX) [file pone.0269068.s013.docx]

**
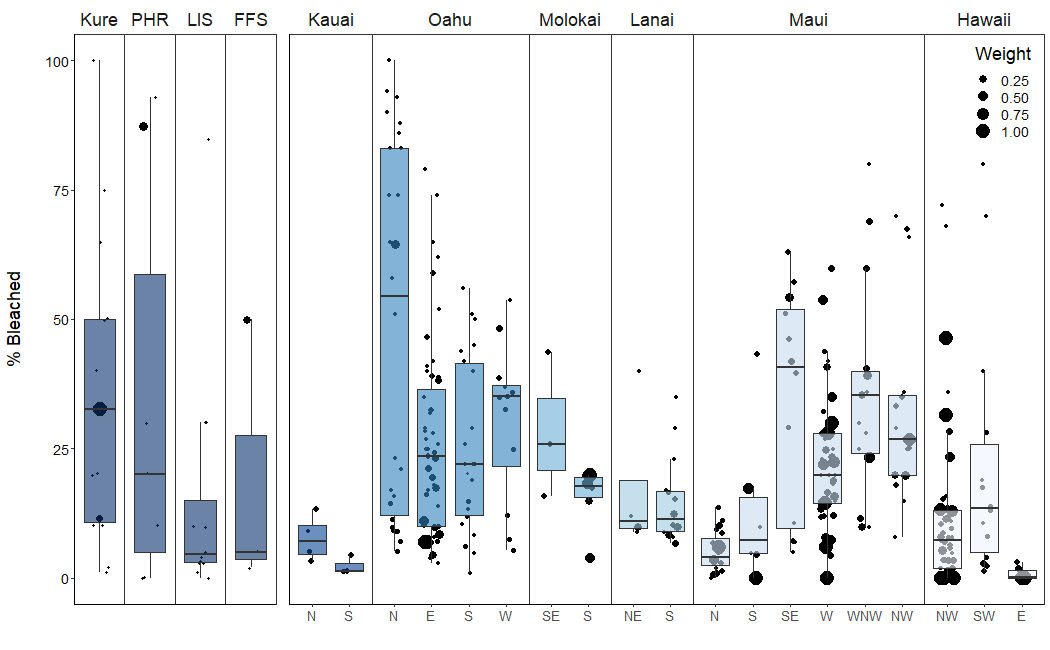
**

**S3 Figure. Box plots of cluster-level observed percent bleached per island (NWHI) or zone (MHI).** Points are sized by weights assigned per cluster. Zones are abbreviated in grey along the x-axis.
