# Supplementary material for: Coral taxonomy and local stressors drive bleaching prevalence across the Hawaiian Archipelago in 2019
Source: PLoS One. 2022 Sep 1;17(9):e0269068. doi: 10.1371/journal.pone.0269068 (PMC9436070; doi:10.1371/journal.pone.0269068)
Supplement: S5 Fig — Points represent individual clusters, with shape indicating depth bin. Zones are denoted by white polygons. (DOCX) [file pone.0269068.s015.docx]

**
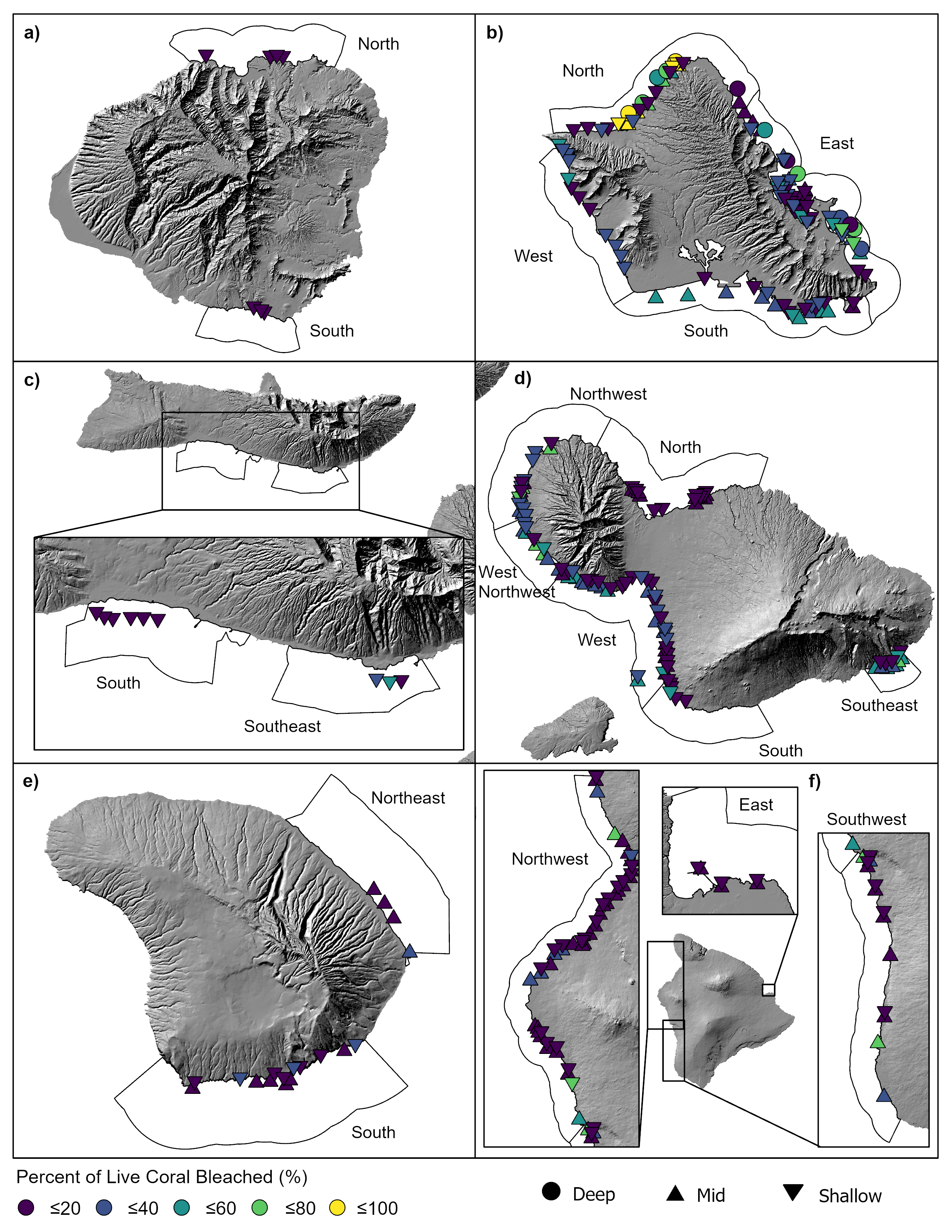
** **S5 Figure. Mean percent bleached per cluster across the main Hawaiian Islands in 2019.** Points represent individual clusters, with shape indicating depth bin. Zones are denoted by white polygons.
