# Supplementary material for: Coral taxonomy and local stressors drive bleaching prevalence across the Hawaiian Archipelago in 2019
Source: PLoS One. 2022 Sep 1;17(9):e0269068. doi: 10.1371/journal.pone.0269068 (PMC9436070; doi:10.1371/journal.pone.0269068)
Supplement: S6 Fig — Points are sized by weights assigned per cluster. (DOCX) [file pone.0269068.s016.docx]

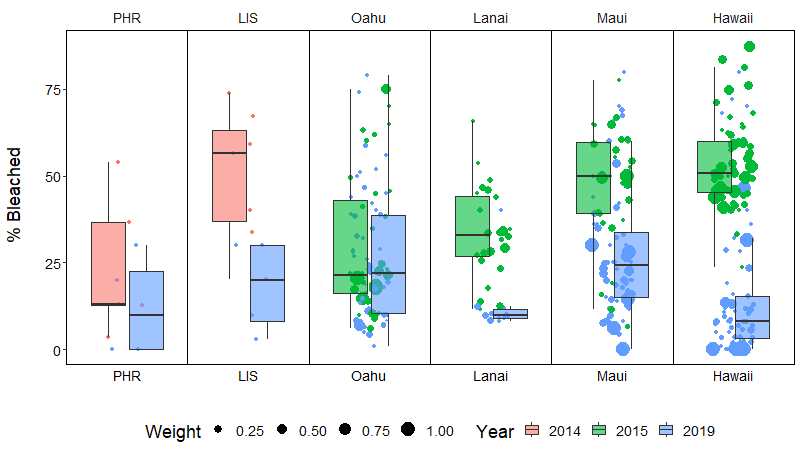


**S6 Figure. Box plots of cluster-level percent bleached (%) per island during the 2014 (NWHI) or 2015 (MHI) bleaching event and the 2019 bleaching event (both regions)**. Points are sized by weights assigned per cluster.
