# Supplementary material for: Coral taxonomy and local stressors drive bleaching prevalence across the Hawaiian Archipelago in 2019
Source: PLoS One. 2022 Sep 1;17(9):e0269068. doi: 10.1371/journal.pone.0269068 (PMC9436070; doi:10.1371/journal.pone.0269068)
Supplement: S7 Fig — Points are sized by weights assigned per cluster. (DOCX) [file pone.0269068.s017.docx]

*
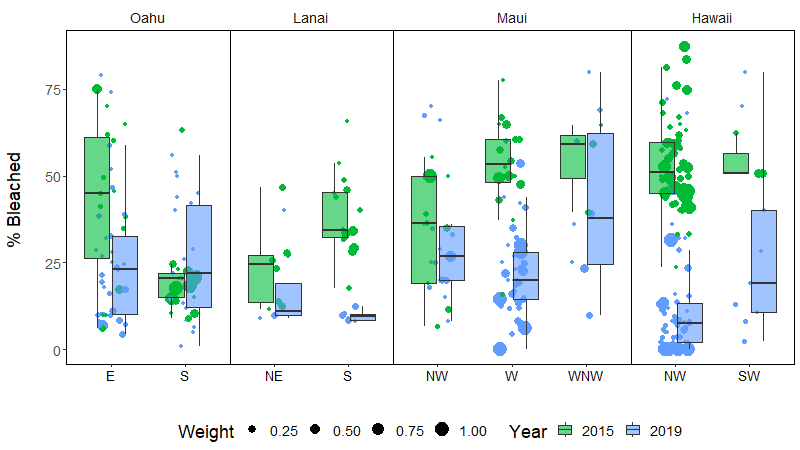
*

**S7 Figure. Box plots of cluster-level percent bleached per zone in the MHI during the 2015 and 2019 bleaching events.** Points are sized by weights assigned per cluster.
