# Supplementary figures and images for: Coral taxonomy and local stressors drive bleaching prevalence across the Hawaiian Archipelago in 2019
Source: PLoS One. 2022 Sep 1;17(9):e0269068. doi: 10.1371/journal.pone.0269068 (PMC9436070; doi:10.1371/journal.pone.0269068)

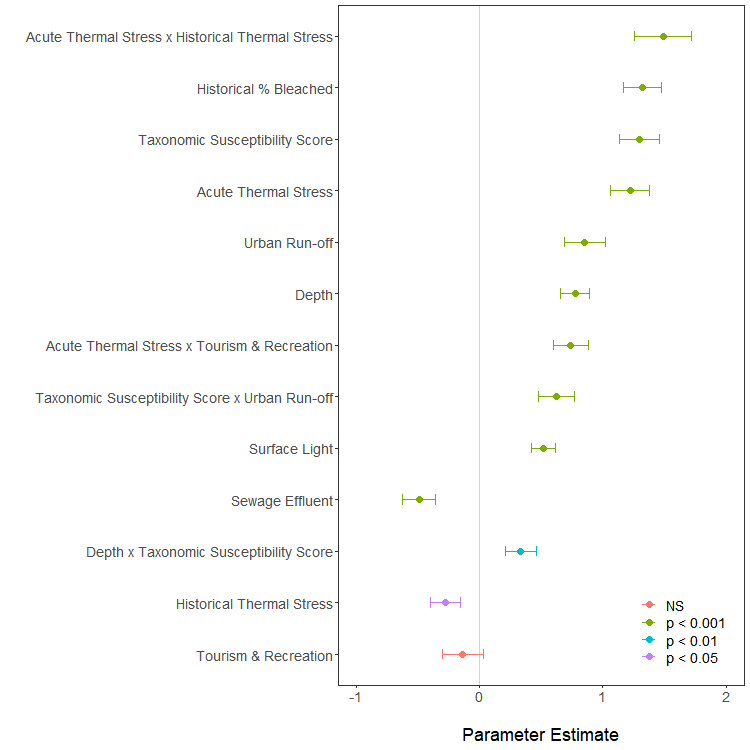


**S8 Figure. Parameter estimates of best-fit model (± SE).** See S8 Table for variable descriptions.

Supplement: S8 Fig — See S8 Table for variable descriptions. (DOCX) [file pone.0269068.s018.docx]
