# Supplementary material for: Coral taxonomy and local stressors drive bleaching prevalence across the Hawaiian Archipelago in 2019
Source: PLoS One. 2022 Sep 1;17(9):e0269068. doi: 10.1371/journal.pone.0269068 (PMC9436070; doi:10.1371/journal.pone.0269068)
Supplement: S9 Fig — Points represent the mean predicted bleaching (%) determined per model perturbation, when each variable was increased/decreased by 1 SD, with all other variables held at original observed values. Error bars represent standard error of the mean. Black line denotes mean of predicted % bleached by unperturbed model. See S8 Table for variable descriptions. (DOCX) [file pone.0269068.s019.docx]

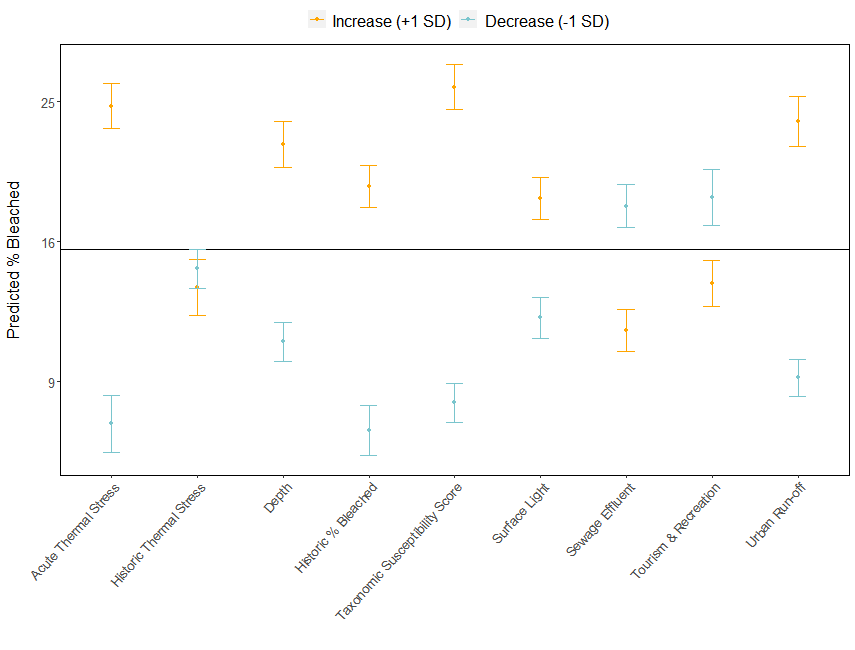


**S9 Figure. Model perturbation plots.** Points represent the mean predicted bleaching (%) determined per model perturbation, when each variable was increased/decreased by 1 SD, with all other variables held at original observed values. Error bars represent standard error of the mean. Black line denotes mean of predicted % bleached by unperturbed model. See S8 Table for variable descriptions.
